# Chemotactic Response and Adaptation Dynamics in *Escherichia coli*

**DOI:** 10.1371/journal.pcbi.1000784

**Published:** 2010-05-20

**Authors:** Diana Clausznitzer, Olga Oleksiuk, Linda Løvdok, Victor Sourjik, Robert G. Endres

**Affiliations:** 1 Division of Molecular Biosciences, Imperial College London, London, United Kingdom; 2 Centre for Integrated Systems Biology at Imperial College, Imperial College London, London, United Kingdom; 3 Zentrum für Molekulare Biologie der Universität Heidelberg, DKFZ-ZMBH Alliance, Heidelberg, Germany; University of Illinois at Urbana-Champaign, United States of America

## Abstract

Adaptation of the chemotaxis sensory pathway of the bacterium *Escherichia coli* is integral for detecting chemicals over a wide range of background concentrations, ultimately allowing cells to swim towards sources of attractant and away from repellents. Its biochemical mechanism based on methylation and demethylation of chemoreceptors has long been known. Despite the importance of adaptation for cell memory and behavior, the dynamics of adaptation are difficult to reconcile with current models of precise adaptation. Here, we follow time courses of signaling in response to concentration step changes of attractant using *in vivo* fluorescence resonance energy transfer measurements. Specifically, we use a condensed representation of adaptation time courses for efficient evaluation of different adaptation models. To quantitatively explain the data, we finally develop a dynamic model for signaling and adaptation based on the attractant flow in the experiment, signaling by cooperative receptor complexes, and multiple layers of feedback regulation for adaptation. We experimentally confirm the predicted effects of changing the enzyme-expression level and bypassing the negative feedback for demethylation. Our data analysis suggests significant imprecision in adaptation for large additions. Furthermore, our model predicts highly regulated, ultrafast adaptation in response to removal of attractant, which may be useful for fast reorientation of the cell and noise reduction in adaptation.

## Author Summary

Bacterial chemotaxis is a paradigm for sensory systems, and thus has attracted immense interest from biologists and modelers alike. Using this pathway, cells can sense chemical molecules in their environment, and bias their movement towards nutrients and away from toxins. To avoid over- or understimulation of the signaling pathway, receptors adapt to current external conditions by covalent receptor modification, ultimately allowing cells to chemotax over a wide range of background concentrations. While the robustness and precision in adaptation was previously explained, we quantify the dynamics of adaptation, important for cell memory and behavior, as well as noise filtering in the pathway. Specifically, we study the intracellular signaling response and subsequent adaptation to concentration step changes in attractant chemicals. We combine measurements of signaling in living cells with a dynamic model for strongly coupled receptors, even including the effects of concentration flow in the experiment. Using a novel way of summarizing time-dependent data, we derive a new adaptation model, predicting additional layers of feedback regulation. As a consequence, adaptation to sudden exposure of unfavorable conditions is very fast, which may be useful for a quick reorientation and escape of the cell.

## Introduction

Cells are able to sense and respond to various external stimuli. To extend the working range of their sensory pathways, biochemical mechanisms allow for adaptation to persistent stimulation, resulting in only a transient response. The dynamics of adaptation are important as they often represent the cells' memory of previous environmental conditions, directly affecting cellular behavior [1]–[7]. Fast adaptation means that cells stop responding and that their biochemical pathways quickly “forget” the stimulus. In contrast, slow adaptation leads to long-lasting effects in the cells. The dynamics of adaptation are often difficult to understand in detail, since they emerge from multiple, simultaneously occurring processes. In higher organisms, adaptation is best documented in the insect and vertebrate visual system, where multiple mechanisms adjust the receptor sensitivity to ambient light levels. For instance, phototransduction in the vertebrate eye involves up to nine different mechanisms for adaptation [8]. However, even in the well-characterized chemotaxis sensory system in *Escherichia coli* [9]–[13], adaptation, in particular its molecular mechanism and dynamics, is not well understood. This constitutes a huge deficit as there has recently been immense interest in the chemotactic behavior of bacteria [14]–[18] and noise filtering [17], [19], [20]. Here, we use adaptation time-course data from *in vivo* fluorescence resonance energy transfer (FRET) measurements and quantitative modeling to address this problem.

The chemotaxis pathway in *E. coli* allows cells to sense chemicals and to swim towards more favorable environments by successive periods of straight swimming (running) and random reorientation (tumbling). Transmembrane chemoreceptors, including the highly abundant Tar and Tsr receptors, cluster at the cell poles and act as “antennas” to detect various attractants (e.g. certain amino acids and sugars) and repellents (e.g. certain metal ions) with high sensitivity [21]. Receptors activate an intracellular signaling pathway, which results in the phosphorylation of diffusible response regulator CheY (CheY-P) via kinase CheA. CheY-P binds to the flagellated rotary motors and induces tumbling. For details of the pathway see the Supplementary Text S1. The interactions between different proteins in the chemotaxis pathway during signaling have been well characterized. Specifically, FRET measurements on the response regulator CheY-P and its phosphatase CheZ have elucidated the signaling in the chemotaxis pathway [22]–[24].

Adaptation in *E. coli* is based on reversible methylation and demethylation of receptors at specific modification sites, catalyzed by enzymes CheR and phosphorylated CheB (CheB-P), respectively. Tar and Tsr receptors have four major modification sites. In addition, the Tsr receptor has two minor modification sites which are methylated less strongly [25]. Receptor modification regulates the receptor activity and provides a recording of experienced concentration changes [16], [26], [27]. As a consequence, the rate of tumbling was found to adapt precisely for different ligand concentrations [28], [29]. To achieve the return of the receptor activity to its pre-stimulus value, receptor activity-dependent phosphorylation of CheB provides a negative feedback on the receptor activity. In addition, the rates of methylation and demethylation depend on the receptor activity [30]–[32], representing further layers of feedback regulation. To modify receptors, CheR and CheB molecules can bind to a specific tether sequence at the carboxyl-terminus of Tar and Tsr receptors, and act on several nearby receptors, so-called assistance neighborhoods [33]. This is believed to compensate for the low numbers of CheR and CheB (hundreds of molecules) [34], although larger numbers have been reported [35].

Although a lot is known about the components of the chemotaxis pathway, several open questions remain to be answered in adaptation. (*i*) Despite their importance for cell behavior, memory and noise filtering, the dynamics of adaptation and the methylation level are largely unknown. This is because the methylation level is difficult to measure precisely, relying on quantification of receptor protein and radioactively-labeled methylation substrate (methionine) incorporated into receptors [25], [36]–[38]. So far, only the initial rate of adaptation was inferred from the rate of change in motor bias in response to saturating amounts of added attractant [29]. (*ii*) The molecular mechanism of adaptation, in particular demethylation, remains unclear. While CheR binds strongly to the tether, suggested to increase its concentration in the vicinity of methyl-accepting sites [39], the binding affinity of CheB was found to be very low [40]. Instead, binding of CheB-P to the tether induces an allosteric activation of the receptor, increasing the demethylation rate [40]. Furthermore, while the receptor activity-dependence of the methylation and demethylation rates is believed to be a requirement for robust precise adaptation (see below), it is not known if adaptation is precise at the receptor level. Time-course data from *in vivo* FRET experiments, monitoring receptor activity upon stimulation, is ideally suited to study the adaptation dynamics and address these questions.

Extensive mathematical modeling has described different aspects of the chemotaxis pathway. However, modeling has mainly focused on explaining the initial response to addition of attractant, as well as precise adaptation, i.e. the complete return of the signaling activity to pre-stimulus level long after the stimulus. Briefly, the Monod-Wyman-Changeux (MWC) model was used to successfully describe the signaling of two-state receptor complexes, formed by 10–20 strongly interacting receptor dimers [24], [41]–[44]. In this model, receptor-receptor coupling provides a mechanism for signal amplification and integration. Alternative receptor models are outlined in the *Discussion*. Furthermore, Barkai and Leibler showed that precise adaptation is robust (insensitive to parameter variations in the pathway), if the kinetics of receptor methylation depends only on the activity of receptors and not explicitly on the receptor methylation level or external chemical concentration [45]. Their idea was later extended by others, providing conditions for precision [46], [47], as well as robustness to noise by the network architecture [48] and assistance neighborhoods [42], [49]. Most recently, adaptation to exponential ramps and sinusoidal concentration changes was investigated [20]. However, none of these studies have directly compared to adaptation time-courses from FRET.

**Figure 1:**
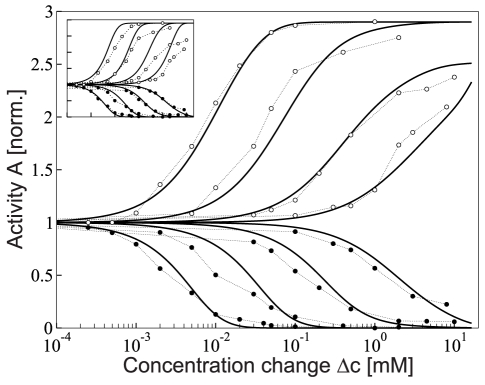
Response of wild-type cells to step changes 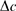 of MeAsp concentration at different ambient concentrations. Dose-response curves: Symbols represent averaged response from FRET data (WT1) after adaptation to ambient concentrations 0, 0.1, 0.5 and 2 mM as measured by Sourjik and Berg [23] (filled and open circles correspond to response to addition and removal of attractant, respectively). Solid lines represent the dynamic MWC model of mixed Tar/Tsr-receptor complexes including ligand rise (addition) and fall (removal), as well as adaptation (receptor methylation) dynamics. (*Inset*) Dose-response curves for MWC model without adaptation dynamics (lines). FRET and receptor complex activities were normalized by adapted pre-stimulus values at each ambient concentration. Squared errors between model and experimental data for the four dose-response curves shown are 0.64 (dynamic MWC model) and 3.95 (static MWC model), respectively. For comparison, fitting to eight addition and removal dose-response curves using 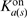, 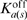, as well as a linear function 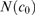 as fitting parameters, yields squared errors 0.83 (dynamic MWC model) and 2.09 (static MWC model), see Supplementary Text S1.

Here, we use *in vivo* FRET data obtained from cells adapted to ambient concentrations of attractant 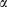-methylaspartate (MeAsp; a non-metabolizable variant of amino acid aspartate) and stimulated in a flow chamber by various concentration step changes [23]. Recording the average initial response amplitudes for each added and, after adaptation, removed concentration step change results in dose-response curves (Fig. 1, symbols). We use a dynamic version of the MWC model, which, in addition to mixed complexes of Tar and Tsr receptors, includes a more detailed description of the adaptation dynamics than used in previous models of chemotaxis. Specifically, we predict multiple layers of feedback regulation during adaptation, especially for demethylation by CheB. In addition, we take into account the kinetics of attractant flow in FRET experiments. This allows us to quantitatively describe dose-response curves (Fig. 1, lines), in particular the observed reduced response amplitudes for removal of MeAsp, which previously could not be explained by the MWC model (*Inset*). To analyze the adaptation dynamics, we use the data collapse, a condensed representation of time courses. This data collapse enables us to evaluate the effect of ligand flow and adaptation imprecision, to infer the kinetics of the receptor methylation level, as well as to efficiently compare adaptation models from the literature to experimental data. Finally, we experimentally test two predictions to validate our adaptation model. We change the adapted receptor activity, and use a non-regulatable CheB mutant to bypass its negative feedback on the receptor activity. Our combined study of experiments and modeling shows that adaptation is relatively imprecise at the receptor level for large stimuli, and that demethylation is more tightly regulated than previously thought. This leads to very short tumbles for sudden occurrences of unfavorable conditions, allowing cells to quickly reorient their swimming direction after a short tumble.

## Results

### Dynamic MWC model for *in vivo* FRET response

Our dynamic MWC model, described in the following, combines the previously used MWC model for receptor signaling by strongly-coupled receptor complexes (denoted here by static MWC model), with the dynamic effects of adaptation by receptor modification, as well as ligand concentration flow. In the static MWC model, mixed receptor complexes composed of Tar (aspartate receptor) and Tsr (serine receptor, which also binds aspartate with lower affinity) are considered in their *in vivo* ratio. Using a two-state assumption, the activity of a receptor complex is given by its probability to be in *on* (active), which depends on the free-energy difference 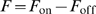 between its *on* and *off* (inactive) state [41], [43],
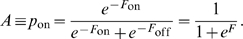
(1)This free-energy difference, 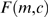, is determined by two contributions, one from methylation (in terms of receptor methylation level 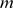) favoring the *on* state, and one from attractant binding at MeAsp concentration 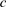 favoring the *off* state. The free-energy difference 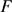 also depends on several parameters such as free-energy difference per added methyl group, the number 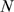 of receptor dimers in a complex, as well as the ligand dissociation constants 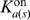 and 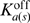 for Tar (Tsr) receptors in their *on* and *off* states, respectively. Most of these parameters were determined previously (see *Materials and Methods*). Similar free-energy based two-state models were recently used to describe clustering of ion channels [50] and small GTPases in eukaryotic cells [51]. In the new dynamic MWC model, we include the effects of variable receptor complex sizes, adaptation dynamics, and MeAsp concentration flow on the initial response to concentration changes.

**Figure 2 pcbi-1000784-g002:**
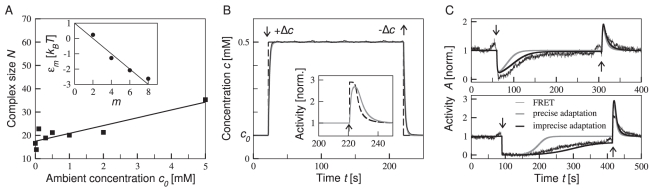
Model ingredients. (A) Size of adapted receptor complex 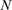 (total number of Tar and Tsr receptors per complex) as function of ambient concentration 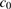 (corresponding to adapted methylation level 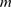). Individual complex sizes (symbols) were obtained by fitting MWC model to dose-response curves for addition of MeAsp. These values were fitted by a linear function (line). (A *Inset*) Energy contribution to receptor complex free energy from methylation level 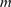 per receptor dimer. Shown are fitting parameters for Tar receptors from [41] (symbols), as well as linear fit 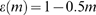 (in units of 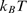 with 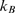 the Boltzmann constant and 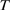 absolute temperature). (B) Profile of concentration step change as measured experimentally using fluorescent marker (solid black line) [23], exponential fit used in dynamic MWC model for WT1 MeAsp profile (gray line; rate constants 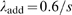 and 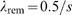), and perfect step change (black dashed line). Addition and removal times are marked by arrows. (B *Inset*) Response of mixed receptor complex to MeAsp removal for perfect (black dashed line) and exponentially fitted step change (gray line). (C) Typical time courses of receptor complex activity in response to two different MeAsp concentration step changes, 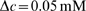 (top) and 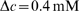 (bottom), at ambient concentration 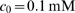. Experimental FRET measurement (thin black line), as well as dynamic MWC model for precise (gray lines) and imprecise adaptation (black lines; 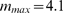 and 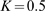). FRET and receptor complex activities were normalized by adapted pre-stimulus values before addition of MeAsp.

The dependence of the receptor complex size on the ambient concentration and hence methylation level was determined as follows: First, the receptor complex size was obtained for each ambient concentration using a least-squares fit to addition dose-response curves (see Fig. 2A and *Materials and Methods*). Consistent with previous modeling results, we find that the receptor complex size increases with increasing ambient concentration [41], [52]. As the simplest assumption, we used a linear relationship between receptor complex size and ambient concentration (Fig. 2A), allowing us to interpolate the receptor complex size for removal dose-response curves. Analyzing the signaling pathway of *E. coli*, we also found the phosphorylation reactions are sufficiently fast to assume that concentrations of phosphorylated (and unphosphorylated) proteins are in quasi-steady state. Furthermore, the concentrations of activated proteins are approximately proportional to the receptor complex activity. Both these conditions allow us to use the receptor complex activity as a substitute for the down-stream activity measured by FRET reducing the number of model parameters for fitting to data greatly (see Supplementary Text S1). This approximation was also used in previous work, but was never explicitly tested [41]–[43].

Adaptation occurs on a similar time scale as the kinetics of the MeAsp concentration flow. In experiments, changes in MeAsp concentration are established over several seconds, due to the finite flow speed and mixing effects in the flow chamber. In our model, we assume exponentially rising and falling concentration changes upon addition and removal in line with previous measurements by Sourjik and Berg (Fig. 2B) [23]. Adaptation is mediated by methylation and demethylation enzymes CheR and CheB, respectively. The process is described by the kinetics of the average receptor methylation level 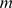 in a receptor complex,
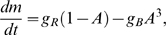
(2)where the adapted receptor-complex activity 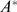 is determined by the steady-state condition 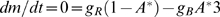. According to our model, receptors are methylated when the complex is inactive, and demethylated when it is active. Furthermore, we postulate a very strong sensitivity of the demethylation rate on activity, possibly due to cooperativity of CheB-P molecules. This mechanism explains the strong asymmetry, which is observed in experimentally measured time courses (cf. Fig. 2C) where adaptation of inactive receptors (methylation) is slow compared to the rapid adaptation of active receptors (demethylation). Hence, during a concentration step change the initial response amplitude of receptor complexes is reduced by simultaneous adaptation, which is particularly important for removal of concentration (see Fig. 2B *Inset*). Note that the asymmetry between slow adaptation of inactive and active receptors, respectively, cannot simply be changed by adjusting the rate constants of methylation and demethylation individually, since they are constrained by the adapted activity 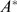. For details of this adaptation model see *Materials and Methods*, and for a potential molecular mechanism of demethylation, see *Discussion*.

Experimental dose-response curves (Fig. 1, symbols) describe the initial response of adapted wild-type cells to sudden changes (addition and removal) in MeAsp concentration [23]. These responses are taken from time courses measured by *in vivo* FRET (cf. Fig. 2). Additional, previously unpublished dose-response curves are provided in the Supplementary Text S1. For details of the experiments see *Material and Methods*. Our dynamic MWC model, which includes the effects of adaptation and MeAsp flow, quantitatively describes the experimental dose-response curves. Specifically, adaptation leads to a non-saturated response for large MeAsp step changes 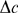 at high ambient concentrations, which is not seen in the static MWC model without adaptation dynamics (Fig. 1 *Inset*). Note, however, that responses eventually do saturate according to the dynamic MWC model for even larger concentration step changes due to the presence of Tsr receptors (at 0.5 mM ambient for 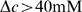; not shown). The dynamic MWC model describes the dose-response data significantly better than the static MWC model, as indicated by their overall squared errors in the caption of Fig. 1, as well as residual errors detailed in the Supplementary Text S1. The receptor-complex activity, as well as FRET data were normalized by their adapted pre-stimulus values at ambient concentration to compare model and experimental data (see *Materials and Methods*).

### Data collapse of time courses for adaptation dynamics

**Figure 3:**
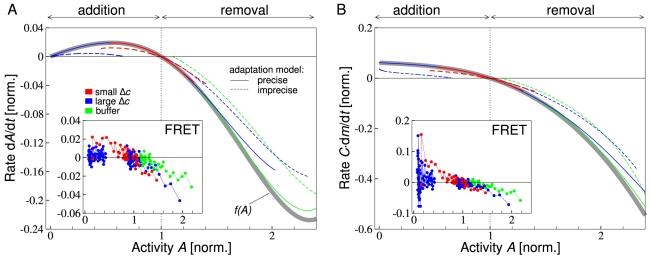
Adaptation dynamics as function of receptor activity for WT1 at ambient concentration 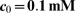 for addition and subsequent removal of small (red lines and symbols) and large (blue lines and symbols) MeAsp concentration step changes, as well as removal of MeAsp back to zero ambient concentration (buffer; green lines and symbols). (A) Rate of change of receptor complex activity 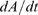 as occurs during adaptation. Thick gray line is the analytical result from the dynamic MWC model, where activity change is purely from adaptation (methylation) 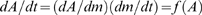. Colored lines show results from simulated time series for small (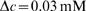) and large (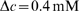) concentration step changes in MeAsp concentration, with activity dynamics recorded starting 10 s after the onset of concentration step change. Precise (solid lines), as well as imprecise adaptation (dashed lines; 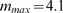 and 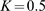) are considered. (A *Inset*) Rate of FRET activity change from experimental time-course data. Small (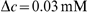) and large (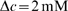) concentration step changes. (B) Rate of change of the methylation level 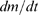 corresponding to panel A (normalized by adapted activity 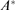 and 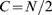, where 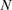 is the receptor complex size). Effective rate of change of methylation level for all time courses is obtained by 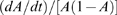. (B *Inset*) Effective rate of change of methylation level from experimental time-course data. FRET and receptor complex activities, as well as activity rate changes were normalized by adapted pre-stimulus activities at ambient concentrations before addition of MeAsp.

The short-term behavior in the time-course data (Fig. 2C) is essential in deriving our adaptation model, used to quantitatively describe dose-response curves (Fig. 1). Can our adaptation model also describe the long-term behavior in the time-course data, and hence the complete adaptation dynamics? Our model for precise adaptation predicts that the observable rate of activity change is given by
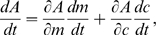
(3)where the rate of change of the methylation level 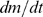 is described by Eq. 2, and 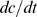 is the rate of change of the MeAsp concentration. After a concentration step change, the MeAsp concentration is constant with 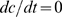, and the rate of activity change is given by
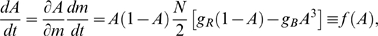
(4)where we used that 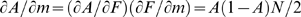 (see *Material and Methods*). Hence, the rate of activity change is a function 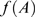 of the activity only, independent of ligand concentration and receptor methylation level (except for the minor dependence of the receptor complex size on the ligand concentration, see Supplementary Text S1). This predicts a data collapse of all adaptation time courses, independent of the duration, size and number of concentration step changes, onto a single curve 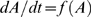 (Fig. 3A, thick gray line). This non-monotonous function of the activity has three fixed points: the adapted activity 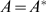, where methylation and demethylation rates exactly balance each other, as well as 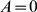 and 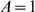, where the receptor complex activity is saturated in the *off* and *on* state, respectively. Figure 3A *Inset* shows the experimental rate of activity change as extracted from our quantitative time-course data from FRET for different concentration step changes at an ambient concentration. We observe that, in contrast to the prediction of the model, the rate of activity change depends on the magnitude of the concentration step changes. For addition of large concentration step changes (blue symbols), the rate is reduced and the activity stays below the pre-stimulus value. Furthermore, for total removal of MeAsp concentration (replacement with buffer medium, green symbols), the magnitude of the rate is reduced and the activity remains above the pre-stimulus value.

To explain the deviations from the predicted data collapse, we consider the effects of MeAsp flow and imprecise adaptation in our model. According to Eq. 3, each of the two effects contribute independently to the rate of activity change. First, we include the MeAsp flow for concentration step changes as described, and simulate time courses based on the precise adaptation model (Fig. 3A, solid lines). We find that in the demethylation regime (negative rate of activity change), the kinetics of concentration step removal gives rise to minor deviations from the curve 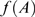 in qualitative agreement with experiment. However, in the methylation regime (positive rate of activity change), unlike the experimental data, all time courses lie accurately on the 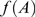 curve. Next, we consider imprecise adaptation, i.e. the incomplete return of the activity to pre-stimulus level, which is apparent in the time courses (Fig. 2C and Supplementary Text S1 for quantification). In our model for imprecise adaptation, Eq. 7 in *Materials and Methods*, the kinetics of the methylation level 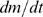 depends explicitly on the receptor methylation level, which leads to significant deviations from the data collapse (Fig. 3A, dashed lines). Adaptation after addition of increasing concentration step changes results in a reduced adapted receptor complex activity (adapted activity after removal is always the same as the concentration is the ambient concentration). Total removal of MeAsp concentration (buffer) results in an increased adapted activity. Our imprecise adaptation model is in line with the experimental data, showing that the data collapse is an effective way to compare experimental and theoretical time courses and to quantify the effects of ligand flow and imprecise adaptation. We also studied the effect of changes in receptor-complex size on the data collapse, which we found to be minor for the concentrations considered here (see Supplementary Text S1). In addition to the adaptation dynamics, the data collapse allows us to determine the kinetics of the receptor methylation level, which is difficult to measure directly. Figure 3B shows the rate of change of the methylation level as a function of the receptor complex activity for experimental data, as well as the dynamic MWC model. The data and curves were obtained by dividing the rate of activity change 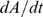 following concentration step changes by 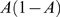. If the activity change is caused only by the adaptation dynamics, this procedure yields a function proportional to the rate of change of the methylation level, 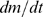. According to our precise adaptation model Eq. 2, the rate of change of the methylation level is a monotonically decreasing function of activity with one steady state, marking the adapted receptor complex activity (Fig. 3B, thick gray line). Corresponding to the rate of activity change in Fig. 3A, the kinetics of ligand flow upon concentration step changes, as well as imprecise adaptation result in deviations from this curve. As before, we mainly find signatures of imprecise adaptation in the experimental data in Fig. 3B *Inset*.

### Comparison of different adaptation models

**Figure 4:**
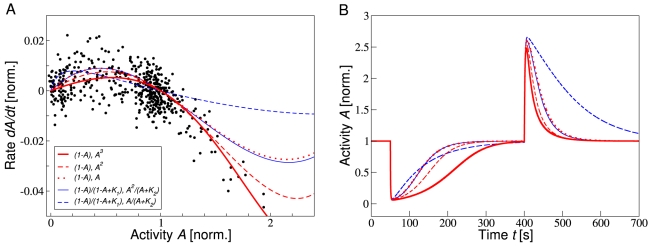
Comparison of different adaptation models. (A) Rate of activity change during adaptation as a function of activity for FRET data (WT1; symbols) and different adaptation models (colored lines). Experimental FRET activity change is measured at ambient concentration 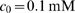 for added and subsequently removed concentration step changes 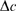 = 0.03, 0.05, 0.1, 0.4 and 2 mM. For the five models, the dependencies of the methylation and demethylation rates on the receptor complex activity 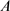 are given in the legend and are explained in the text. Models were fitted to the experimental 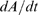 data using a least-squares fit, where the methylation rate constant 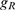 was the only fitting parameter. The demethylation rate 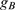 was determined to produce the adapted activity 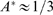. The parameters 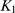 and 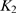 were converted from [17]. (B) Representative time courses for the different models in panel A for a concentration step change 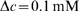 at ambient concentration 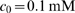. FRET and receptor complex activities, as well as activity rate changes were normalized by adapted pre-stimulus activities at ambient concentrations before addition of MeAsp. Least-squares errors between experimental data and model in panel A are 0.0021 [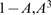], 0.0022 [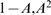], 0.0025 [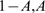], 0.0025 [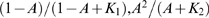], and 0.0036 [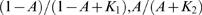].

The data collapse of experimental time courses enables the efficient evaluation of different adaptation models, including our model and other models from the literature (Fig. 4A). All models considered here show precise adaptation with the rates of methylation and demethylation only depending on the receptor complex activity, and the explicit activity dependencies given respectively by the first and second term in the legend of Fig. 4. For instance, the first model (solid red line) is given by Eq. 2. Two classes of models are analyzed here. The first class of models, including our model, is based on a linear activity-dependence of the methylation and demethylation rates for binding of CheR and CheB to inactive and active receptor, respectively. Feedback from the activity-dependent phosphorylation of CheB is accounted for by additional factors of the receptor complex activity. Our model includes cooperative CheB feedback (solid red line), while linear CheB feedback (dashed red line) and no CheB feedback (dotted red line) are considered as well [15], [42], [49], [53]. Another class of models has been proposed, showing ultrasensitivity with respect to CheR and CheB protein levels. In these models, the activity-dependence of the methylation and demethylation rates for enzyme binding is described by Michaelis-Menten kinetics, and linear CheB feedback (solid blue line) and no CheB feedback (dashed blue line) is considered [17]. The last model has the property that the rate of change of methylation level becomes independent of activity around the steady-state, leading to extremely long adaptation times. Details of the alternative adaptation models and the fitting procedure are given in the Supplementary Text S1. While several models are consistent with the experimental data, our model compares most favorably. The ultrasensitive Michaelis-Menten model without CheB feedback seems not to be consistent with the data. Comparing simulated time courses for the different adaptation models in Fig. 4B, our model is best to capture the experimentally observed asymmetry between adaptation to addition and removal of concentration step changes. The quality of fit between the respective models and data is indicated by their least-squares errors in the caption of Fig. 4.

### Predictions

**Figure 5:**
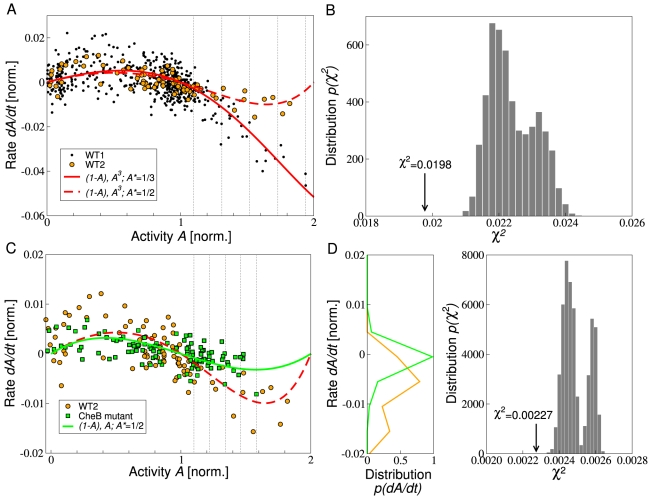
Effects of (A) steady-state activity and (C) CheB regulation by phosphorylation. (A) Black and orange dots correspond to the rate of FRET activity change from experimental time-course data for WT1 (Fig. 4) and for WT2 (addition and subsequent removal of concentration step change 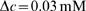 at zero ambient concentration), respectively. Red lines correspond to the predicted rate of activity change 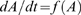 purely from adaptation (solid and dashed lines correspond to steady-state activities 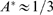 and 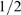, respectively). The methylation rate constant 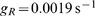 is the same in each case. Dotted lines indicate bins used to quantify the difference between data sets in panel B. (B) Distribution of squared errors 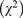 between predicted rate of activity change and experimental data sets for WT1 and WT2, when randomly permuting 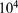 pairs of data points between the data sets, one pair chosen within each bin in panel A. The error is calculated as the sum of errors for each data set (including the permuted data points) against its respective model. The error of the unpermuted data sets is indicated by the arrow. (C) Green squares represent the rate of FRET activity change from experimental time-course data for CheB mutant (addition and subsequent removal of concentration step changes 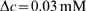 and 0.1 mM at zero ambient concentration). The green line represents the rate of change of receptor complex activity purely from adaptation. Orange dots and red dashed line are the same as in panel A. Dotted lines indicate bins used to quantify the difference between data sets in panel D. (D, left) Distribution of data points of the rate of activity change for activities above 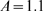 WT2 and CheB mutant data in panel C. (D, right) Distribution of squared errors between predicted rate of activity change and experimental data sets for WT2 and CheB mutant, when randomly permuting 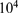 pairs of data points between the data sets, one pair chosen within each bin in panel C. The error is calculated as the sum of errors for each data set (including the permuted data points) against its respective model. The error of the unpermuted data sets is indicated by the arrow.

To further validate our adaptation model, we experimentally tested two predictions. First, in our precise-adaptation model the data collapse depends strongly on the steady-state activity. For instance, increasing the steady-state activity from 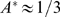 to 1/2 changes the data collapse from the solid to the dashed red line in Fig. 5A. Such an increase in the steady-state activity can be achieved by decreasing CheB expression level, corresponding to a decreasing demethylation rate, at constant CheR expression level. To validate this prediction, a different wild-type strain (WT2) was created, in which CheB expression was induced from a plasmid, while all other chemotaxis proteins were expressed as before (WT1). The steady-state activity was estimated to be 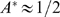 (compared to 1/3 in WT1). For details of the strains, see *Materials and Methods*. The data collapse (Fig. 5A, orange circles) corresponds well to the predicted curve (dashed red line). Second, the activity-dependence of the demethylation rate is diminished according to Eq. 6 when considering adaptation without feedback through activity-dependent CheB phosphorylation, while keeping the steady-state activity constant (Fig. 5C, green line). To validate this prediction, a mutant strain was created, which contains non-regulatable CheB with about 10 percent of CheB-P activity. The CheB expression level was increased to produce the kinase activity of WT2 (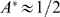). All other chemotaxis proteins are expressed as in WT2 cells. We find that the experimental rate of FRET-activity change from time-course data (green squares) is consistent with this prediction.

The statistical significance for each of the two predictions (Fig. 5A and C) was tested as follows: For each prediction, we randomly permuted a number of data points from the control experiment and the prediction-testing experiment. Then we calculated the distribution of squared errors between the rates of activity change from the model and FRET measurement for the permuted data sets (Fig. 5 B and D). For four permuted pairs of data points the error is always above the error for the unpermuted data sets (Fig. 5). For fewer permutations the error lies at the lower bound of the distribution (not shown). This confirms that the control and prediction-testing data sets are significantly different and match our model.

## Discussion

Sensing and adaptation are fundamental biological processes, enabling cells to respond and adjust to their external environment. Adaptation extends the range of stimuli a sensory pathway can respond to, while its dynamics determines how long a stimulus will affect the cell's behavior. In this work, we developed a model to quantitatively describe experimental dose-response curves, as well as time courses of chemotaxis signaling in adapting wild-type cells. Our model includes (*i*) the signaling activity of two-state mixed chemoreceptor complexes in response to added or removed attractant concentration step changes based on the Monod-Wyman-Changeux model, (*ii*) the kinetics of the ligand concentration in the flow chamber, and (*iii*) a detailed mechanism for adaptation, including multiple layers of feedback regulation and imprecise adaptation. In particular, we find that the finite ligand flow speed and fast, activated demethylation explains for the first time the gradually reduced amplitudes in removal dose-response curves for increasing ambient concentrations (Fig. 1). Our adaptation model introduces a strong receptor-activity dependence of the demethylation rate, and hence is able to reproduce the observed asymmetry of slow adaptation to addition of attractant and fast adaptation to removal of attractant (Fig. 2C). Such dynamics yields long runs up the gradient and short tumbles, sufficient for random reorientation of the cell and escape from potential toxins. Furthermore, this strong activity dependence has the additional benefit of reducing the fluctuations in receptor methylation level introduced by the adaptation mechanism itself. We found for the total variance of the receptor-complex methylation level 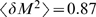 compared to 2 for a previous model for precise adaptation with weaker activity dependence (details of the calculation can be found in the Supplementary Text S1). This is because a fluctuation in the receptor methylation level leads to an increased change in activity and hence increased rate to return to the adapted activity.

Our model for precise adaptation predicts the data collapse of adaptation time-courses, allowing the convenient study of the adaptation dynamics (Fig. 3A). Specifically, the data collapse allows to evaluate the effects of ligand flow and adaptation dynamics, as well as imprecise adaptation. We found that adaptation to large concentration step changes is significantly imprecise (see Supplementary Text S1 for further details). We also extracted the kinetics of the receptor methylation level 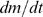 from experimental time courses from the data collapse (Fig. 3B), which is difficult to measure directly when relying on the quantification of the receptor methylation level using standard biochemical methods [25], [36]. According to our model, the activity-dependence of the receptor methylation level is a monotonously decreasing function of the receptor complex activity. Ultimately, this kinetics determines the compromise between long memory of previous concentrations and quick recovery for sensing new concentration changes [14]. Furthermore, we experimentally tested two predictions to validate our adaptation model. We analyzed the effect on the adaptation dynamics when changing the adapted receptor activity, as well as introducing a non-regulatable CheB mutant to remove the negative feedback from phosphorylation of CheB by the kinase CheA. In both cases, our model is consistent with experimental measurements (Fig. 5), supporting the finding of multiple layers of feedback regulation in adaptation.

While the MWC model is relatively well established [24], [41]–[44], we also considered alternative models for receptor signaling. These include a phase-separation model with mixed complexes separating into homogeneous complexes of Tar and Tsr at high ambient concentrations, as well as a lattice model with finite coupling between neighboring receptors (see Supplementary Text S1). Lattice models were previously suggested [54], [55], including a lattice formed by coupled CheA molecules [56], but were found to be inconsistent with FRET data [57]. We found that the dynamic MWC model describes dose-response curves far better than the alternative receptor signaling models investigated, particularly the reduced response amplitudes upon removal of attractant. Furthermore, the data collapse we introduced in this paper enabled us to compare different adaptation models proposed in the literature with FRET time-course data (Fig. 4). We found that while several models are consistent with the data, our model compared most favorably with the data.

We chose a simple model for adaptation with very few fitting parameters to explain the observed asymmetry in adaptation time-courses, i.e. slow adaptation to addition and fast adaptation to removal of attractant. Compared to the static MWC model, there are minor discrepancies between our model and experimental addition dose-response curves (Fig. 1). However, these can be rectified by refitting the dynamic MWC model by adjusting adaptation rates and receptor complex size simultaneously (see Supplementary Text S1), or by choosing an adaptation model with a more complex activity dependence. It should also be noted that adaptation rates needed to accurately describe dose-response curves are larger than those found when fitting the adaptation dynamics to the data collapse. This discrepancy may in part be due to using only a single set of experimental data for the data collapse, while dose-response curves were averaged over at least three sets. In addition, more complex processes not taken into account in our simple adaptation model, e.g. limited supply of substrate (methionine) for methylation, or the binding and unbinding kinetics of ligand, may be important for describing the dynamics.

Although our adaptation model is independent of biochemical details, our predicted fast demethylation at high activities may be due to cooperativity of two CheB-P molecules. According to *in vitro* experiments, CheB-P binding to the carboxyl-terminus of chemoreceptors induces an allosteric activation of the receptor, increasing the demethylation rate [40]. However, in contrast to CheR, CheB-P binds only weakly to the tether [40]. Reconciling these two observations, it is conceivable that two CheB-P molecules are necessary for efficient demethylation at high activities: one CheB-P molecule may bind to a tether to allosterically activate a group of receptors (assistance neighborhood), while another CheB-P molecule demethylates the receptors in the neighborhood. As two CheB-P molecules are not required to bind to the same receptor, this mechanism is not contradicted by the small number of CheB molecules in a cell. An alternative, simpler mechanism for cooperativity is dimerization of CheB-P molecules, which, however, has not been observed experimentally [22], .

Our adaptation model likely also applies to attractants other than MeAsp, since the dynamics of adaptation only depend on the activity of receptor complexes, independent of the details of external ligand concentration. According to the MWC model, different attractants (or their mixture) are integrated at the level of the free-energy of a receptor complex, which determines its activity. However, the imprecision of adaptation we found in FRET time courses at large MeAsp concentrations is in contrast to earlier experiments, which showed that the frequency of tumbling adapts precisely to aspartate, but not serine [28], [29]. The imprecision in adaptation to serine is readily explained if the number of Tsr receptors is larger than the number of Tar receptors per complex, since the available receptor methylation sites in a complex are more quickly used up in response to serine binding to Tsr receptors [42], [49]. However, the ratio of Tar and Tsr per complex is strongly dependent on the growth conditions, making a definitive conclusion difficult [59]. Future experiments may show if the imprecision observed in adaptation to MeAsp in FRET is reflected also in the tumbling frequency, or if imprecise adaptation is compensated for in order to yield perfect adaptation at the behavioral level.

## Materials and Methods

### Strains

Two different wild-type strains of *E. coli* were used. Wild-type strain 1 (WT1) is VS104 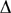
(cheY cheZ) that expresses the FRET pair consisting of CheY-YFP (YFP; yellow fluorescent protein) and its phosphatase CheZ-CFP (CFP; cyan fluorescent protein) from a pTrc-based plasmid pVS88, which carries pBR replication origin and ampicillin resistance and is inducible by isopropyl 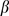-D-thiogalactoside (IPTG) [23]. Wild-type strain 2 (WT2) is VS124 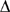
(cheB cheY cheZ) transformed with pVS88 and pVS91, which carries pACYC replication origin and chloramphenicol resistance and encodes wild-type CheB under control of pBAD promoter inducible by L-arabinose. The CheB-mutant strain is VS124 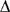
(cheB cheY cheZ) transformed with pVS88 and pVS97, which is identical to pVS91 except it encodes the non-regulatable CheB^D56E^. The D56E mutation was introduced into CheB by PCR. It prevents CheB phosphorylation, but allows protein to retain basal level of activity. Cells were grown at 275 rpm in a rotary shaker to mid-exponential phase (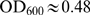) in tryptone broth (TB) medium supplemented with 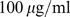 ampicillin, 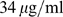 chloramphenicol, and 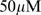 IPTG. WT and CheB mutant strains were induced by 0 and 0.0003% arabinose, respectively, to produce comparable kinase activity (as assessed by the change in the level of FRET upon saturating stimulation). The CheB protein level was estimated using Western blots with CheB antibodies, and was at approximately 0.5-fold (WT2) and approximately 5-fold (CheB^D56E^ mutant) the native level of CheB.

### FRET measurements

The experimental procedures follow those detailed by Sourjik and Berg [23]. Cells were tethered to a cover slip, and placed in a flow chamber. Cells were subject to a constant fluid flow of buffer or MeAsp at indicated concentration (flow speeds 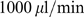 for WT1, and 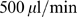 for WT2 and CheB mutant, respectively). Concentration step changes were achieved by abruptly switching between buffer and MeAsp, or different MeAsp concentrations. Fluorescence resonance energy transfer (FRET) between excited donor, CheZ-CFP, and acceptor, phosphorylated CheY-YFP, in a population of 300–500 cells was monitored using an epifluorescence microscopy setup. Emission light from CFP and YFP was collected and their intensity ratio 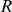 was used to calculate the time-dependent number of interacting FRET pairs of CheZ-CFP and phosphorylated CheY-YFP in the cell population, which reflects the intracellular kinase activity [23]. The number of FRET pairs normalized by its adapted pre-stimulus value (after adaptation to the ambient concentration, but before concentration step changes) was calculated from the ratio 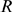 according to 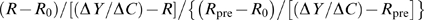 [23]. The parameters 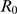 and 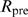 are the ratio for a saturating dose of attractant and the pre-stimulus value, respectively, both of which are measured in each experiment. The fluorescence efficiency ratio 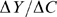 is determined by the experimental setup [60], and was 0.43 (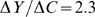) for WT1 (WT2 and CheB mutant) experiments. FRET measurements were taken with a time resolution of 0.2 s (1 s) for WT1 (WT2 and CheB mutant).

### Static MWC model

This model describes the response of adapted mixed receptor complexes to instantaneous MeAsp concentration step changes [24], [43], [44]. According to this model, the activity of a mixed receptor complex is given by 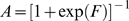, where
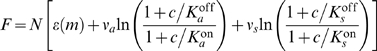
(5)is the free-energy difference between the *on* and *off* states of the complex. The indexes 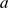 and 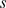 denote Tar and Tsr receptor, respectively. We assumed fractions of Tar and Tsr in a complex according to their wild-type ratio, 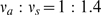. The ligand dissociation constants for MeAsp are 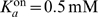, 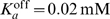, 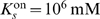, and 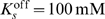 [43]. The free-energy contribution 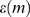 is attributed to methylation, and was recently extracted from dose-response curves for mutants resembling fixed methylation states [41]. We used the interpolating function 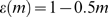 (for data and fit see *Inset* of Fig. 2A). All energies are measured in units of 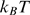 (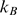 being the Boltzmann constant and 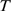 the absolute temperature). Exponential rate constants for the ligand flow were obtained from fits to ligand flow profiles (cf. Fig. 2B), with 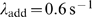 and 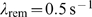 for flow speed 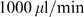, and 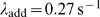 and 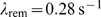 for flow speed 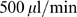. The receptor complex size 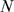 was estimated from least-squares fits to individual addition dose-response curves corresponding to specific ambient concentrations (and therefore adapted methylation levels). Note that complex size for removal may be different for each data point as cells are adapted to the increased concentration after each step change. The complex size grows with ambient concentration [41], [52] in a roughly linear fashion, 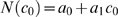 with 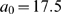 and 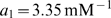. Both, individually fitted 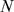 values, as well as the fitting function 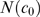, are shown in Fig. 2A. We assumed an adapted receptor complex activity 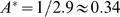 for WT1 as assessed from the maximum and minimum values of the experimental dose-response data in Fig. 1. Steady-state activities for WT2 and CheB mutant were estimated to be 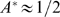. For comparison of model and data, we normalized the receptor-complex activity for WT1, WT2 and CheB mutant by their respective activities when adapted to ambient concentration.

### Precise adaptation

The dynamic MWC model combines the static MWC model with a dynamical model for adaptation. In our model for precise adaptation, the rate of change of the average receptor methylation level 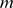 is given by (Eq. 2)
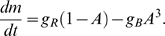
The methylation and demethylation rates do not depend directly on the concentration of MeAsp or the methylation level, only on the receptor complex activity 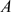. Such dynamics leads to precise adaptation of the receptor complex activity to adapted level 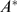 for a constant MeAsp stimulus [42], [45]. This model takes into account the receptor-activity dependence of the methylation and demethylation rates, as well as additional layers of feedback regulation for demethylation by CheB, including the activation of demethylation enzyme CheB by phosphorylation and potential cooperativity between phosphorylated CheB molecules. For Fig. 1–3, we determined the demethylation rate 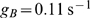 from a least-squares fit to addition and removal dose-response curves (WT1) using the receptor complex size 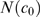 from the static MWC model. The methylation rate 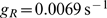 is given by the condition that at steady state (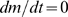) the activity equals 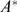. The fit to the data collapse in Fig. 4 resulted in 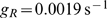 (and 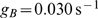), used in Fig. 4 and 5 for WT1. For WT2 in Fig. 5A, we used the same methylation rate constant as for WT1, but adjusted the demethylation rate constant to account for the increased adapted activity 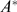. For the CheB mutant in Fig. 5C, the rate of change of the average receptor methylation level 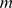 is predicted to be
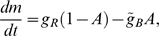
(6)where we assume that the methylation rate is the same as for wild-type cells. The demethylation rate constant 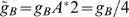 includes the basal activity of non-phosphorylatable CheB. Hence, the only dependence of the demethylation rate on receptor complex activity is due to binding of CheB to active receptors.

### Imprecise adaptation

We incorporate the effect of imprecise adaptation, as suggested by time courses (cf. Fig. 2C), by making methylation and demethylation rates for wild-type cells (WT1) depend on the methylation level [49]

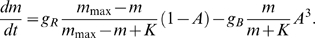
(7)The parameter 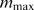 is the maximum number of methylation sites per receptor, 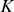 is the lower bound for the number of sites, which need to be available for efficient methylation or demethylation. We use 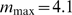 to only allow Tar (not Tsr) receptors to become methylated (the total number of methylation sites of a receptor homodimer being 8). Further, we use 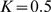 to implement reduced efficiency of methylation or demethylation at a low number of available sites. Figure 2C shows time courses for adaptation to two concentration step changes using the precise and imprecise adaptation model (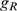 and 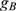 are the same in both models). The imprecise adaptation model fits the time courses shown far better. However, there is a large variability of imprecision seen in different data sets and more experiments are needed to produce a general model of imprecise adaptation.

### Rate of activity change

To calculate the rate of activity change, the time courses for adaptation to step concentration changes were smoothed by averaging every 20 subsequent data points starting approximately 10 s after the step onset. The derivative 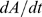 was approximated by the difference quotient.

## Supporting Information

Text S1 Supplementary information.
(0.52 MB PDF)
