# Supplementary material for: Chemotactic Response and Adaptation Dynamics in Escherichia coli
Source: PLoS Comput Biol. 2010 May 20;6(5):e1000784. doi: 10.1371/journal.pcbi.1000784 (PMC2873904; doi:10.1371/journal.pcbi.1000784)
Supplement: Text S1 — Supplementary information. (0.52 MB PDF) [file pcbi.1000784.s001.pdf]

Supplementary Text S1: Chemotactic response and adaptation dynamics in *Escherichia coli*

Diana Clausznitzer[1,2], Olga Oleksiuk[3],
Linda Løvdok[3], Victor Sourjik[3], Robert G. Endres[1,2]*

[1] Division of Molecular Biosciences, Imperial College London, London SW7 2AZ, United Kingdom
[2] Centre for Integrated Systems Biology at Imperial College, Imperial College London, London SW7 2AZ, United Kingdom
[3] Zentrum für Molekulare Biologie der Universität Heidelberg, DKFZ-ZMBH Alliance, Im Neuenheimer Feld 282, 69120 Heidelberg, Germany
∗ E-mail: r.endres@imperial.ac.uk

Contents

1 Review of chemotaxis signaling pathway 1
2 Whole-pathway model 2
2.1 Rescaling of parameters . . . 3
2.2 Steady-state concentrations . . . 4
2.3 Time courses and steady-state assumption . . . 4
3 Additional data and best fit of dynamic MWC model 6
4 Parameters for static and dynamic MWC model 7
5 Effect of receptor complex size on data collapse 8
6 Unsuitable receptor signaling models 9
7 Comparison of different adaptation models 12
8 Analysis of adaptation noise 14
9 Quantification of adaptation imprecision 16

1 Review of chemotaxis signaling pathway

The bacterium *Escherichia coli* chemotaxes by utilizing a biased random walk towards a nutrient source (or away from a toxin source) [1–4]. The swimming path consists of runs, i.e. straight swimming driven by coherent motion of flagella, and tumbles characterized by lack of net movement and random reorientation of the cell.

The molecular components of the chemotaxis signaling pathway and relationships between them are well-characterized [5], and are shown schematically in Fig. 1. Transmembrane chemoreceptors localize predominantly at cell poles, where they form large clusters. There are five different types of chemoreceptors, each with specific sensing capabilities. The two most abundant receptor types, Tar and Tsr, bind respectively the amino acids aspartate (and its non-metabolizable analogue MeAsp) and serine. Tsr also binds aspartate and MeAsp with much lower affinity. Binding of

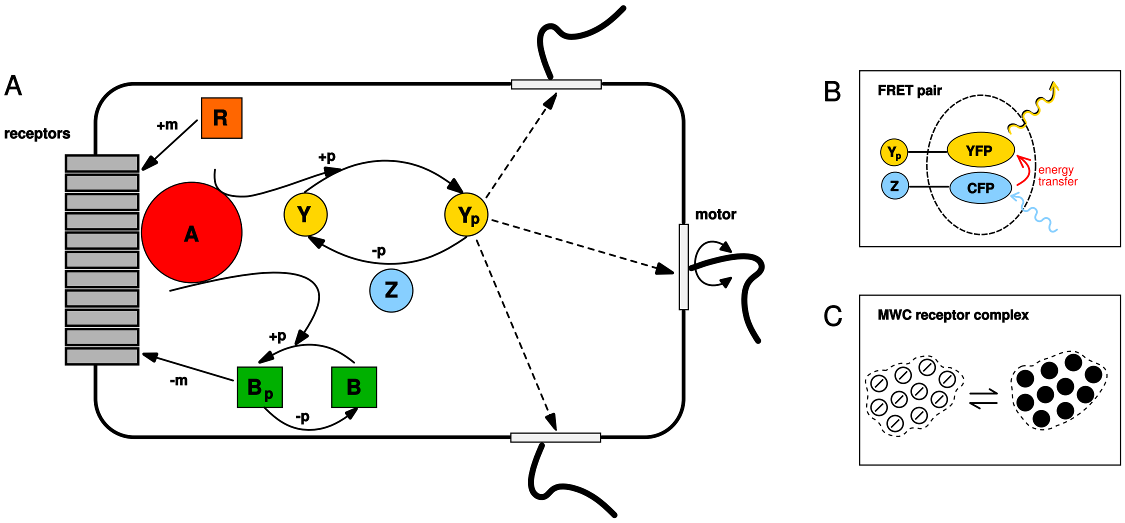

Figure 1: Schematics of chemotaxis signaling and its measurement by FRET. (A) Chemotaxis signaling pathway in *E. coli* from receptors to rotary motors and flagella, including phosphotransfer from CheA to CheY and CheB, CheY-P diffusion to rotary motors, and dephosphorylation of CheY-P by phosphatase CheZ. Adaptation involves receptor methylation by CheR and demethylation by CheB-P. (B) FRET pair CFP/YFP used in experiments by Sourjik and Berg [6]; YFP tags CheY and CFP tags CheZ. CFP is excited by laser light and transfers its energy to YFP during FRET. YFP de-excites by fluorescence. (C) Fast switching between *on* (white discs) and *off* state (black discs) of an MWC receptor complex.

ligand induces signaling by the receptor across the membrane to the kinase CheA. CheA as well as the protein CheW (not shown in Fig. 1) have been suggested to be involved in receptor-receptor coupling and signal integration. When active, CheA autophosphorylates and rapidly passes on a phosphoryl group to its response regulators CheY and CheB. Phosphorylated CheY (CheY-P) diffuses to the rotary motors which drive the cell's flagella. Upon binding to the motors, CheY-P induces a switch in rotary direction resulting in tumbling. CheZ is a phosphatase of CheY-P. Attractant binding reduces the activity of CheA, lowering the concentration of CheY-P in the cell, and therefore suppressing tumbling. In contrast, repellents cause an increase of activity, enhancing tumbling.

Adaptation is mediated by the proteins CheR and CheB. CheR methylates receptors to enhance their signaling activity. Phosphorylated CheB (CheB-P) demethylates receptors, which reduces their activity. During persistent stimulation by a chemical, the combined effect of receptor methylation by CheR and demethylation by CheB-P leads to adaptation of the kinase activity to a steady-state, allowing the sensing of new changes in attractant or repellent concentrations.

2 Whole-pathway model

We consider the following reactions shown in Fig. 1: (1) auto-phosphorylation of CheA and formation of CheA-P (concentrations $[A_p]$) when receptors are active, (2) phosphorylation of CheY and formation of CheY-P ($[Y_p]$), (3) association of CheY-P and CheZ ($[Y_pZ]$), leading to the dephosphorylation of CheY-P and dissociation into CheY and CheZ, and (4) phosphorylation of CheB and formation of CheB-P ($[B_p]$).

Assuming the law of mass-action, our model comprises the following set of ordinary differential equations:

$$\frac{d[A_p]}{dt} = \underbrace{A \cdot k_A \left([A]_{\text{tot}} - [A_p]\right)}_{\text{CheA autophosphorylation}} - \underbrace{k_Y \left([Y]_{\text{tot}} - [Y_p]\right)[A_p]}_{\text{CheY phosphorylation}} - \underbrace{k_B \left([B]_{\text{tot}} - [B_p]\right)[A_p]}_{\text{CheB phosphorylation}} \quad (1)$$

$$\frac{d[Y_p]}{dt} = \underbrace{k_Y\left([Y]_{\text{tot}} - [Y_p]\right)[A_p]}_{\text{CheY phosphorylation}} - \underbrace{k_1\left([Z]_{\text{tot}} - [Y_pZ]\right)[Y_p]}_{\substack{\text{CheY-P/CheZ}\\ \text{association}}} + \underbrace{k_2[Y_pZ]}_{\substack{\text{CheY-P/CheZ}\\ \text{dissociation}}} \tag{2}$$

$$\frac{d[Y_pZ]}{dt} = \underbrace{k_1([Z]_{\text{tot}} - [Y_pZ])[Y_p]}_{\text{CheY-P/CheZ association}} - \underbrace{(k_2 + k_3)[Y_pZ]}_{\substack{\text{CheY-P/CheZ dissociation}\\ \text{and CheY-P dephosphorylation}}} \tag{3}$$

$$\frac{d[B_p]}{dt} = \underbrace{k_B\left([B]_{\text{tot}} - [B_p]\right)[A_p]}_{\text{CheB phosphorylation}} - \underbrace{k_{-B}[B_p],}_{\substack{\text{spontaneous}\\ \text{dephosphorylation of CheB-P}}} \tag{4}$$

where the k_i (with $i = 1, 2, 3, A, B, -B$ and Y) are kinetic rate constants for the individual reactions. The activity A of a receptor complex in Eq. 1 is determined by the MWC model, given by

$$A = \frac{1}{1 + e^F}, \text{ with} \tag{5}$$

$$F = N\left[\epsilon(m) + \nu_a \ln\left(\frac{1 + c/K_a^{\text{off}}}{1 + c/K_a^{\text{on}}}\right) + \nu_s \ln\left(\frac{1 + c/K_s^{\text{off}}}{1 + c/K_s^{\text{on}}}\right)\right], \tag{6}$$

as described in the main text. In addition, we include the adaptation dynamics by methylation and demethylation of receptors, catalyzed by CheR and CheB-P, respectively (cf. Eq. 2 in the main text),

$$\frac{dm}{dt} = \underbrace{g_R(1 - A)}_{\text{methylation by Che-R}} - \underbrace{\hat{g}_B[B_p]^2 A,}_{\text{demethylation by CheB-P}} \tag{7}$$

$$= g_R(1 - A) - g_B A^3 \tag{8}$$

where g_R and $\hat{g}_B$ are effective rate constants, and $\hat{g}_B[B_p]^2 \approx g_B A^2$ as $[B_p]$ is approximately proportional to the receptor complex activity (Fig. 2D). This adaptation model is further explained in the next section. The parameter values we used for the whole-pathway model are listed in Table 1.

2.1 Rescaling of parameters

In order to reduce the number of parameters, we normalize the protein concentrations by their respective total concentrations in the cell, $[A_p] \to [a_p] = [A_p]/[A]_{\text{tot}}$, $[Y_p] \to [y_p] = [Y_p]/[Y]_{\text{tot}}$, $[Y_pZ] \to [y_pz] = [Y_pZ]/[Y]_{\text{tot}}$ and $[B_p] \to [b_p] = [B_p]/[B]_{\text{tot}}$. Furthermore, we rescale the time by the autophosphorylation rate of CheA, k_A, $t \to \tau = k_A \cdot t$, and introduce rescaled rate constants according to $k_1 \to \kappa_1 = k_1[Y]_{\text{tot}}/k_A$, $k_2 \to \kappa_2 = k_2/k_A$, $k_3 \to \kappa_3 = k_3/k_A$, $k_Y \to \kappa_Y = k_Y[A]_{\text{tot}}/k_A$, $k_B \to \kappa_B = k_B[A]_{\text{tot}}/k_A$ and $k_{-B} \to \kappa_{-B} = k_{-B}/k_A$. Overall, this transformation yields dimensionless kinetic variables and parameters by measuring phosphorylated protein fractions in units of total protein concentrations and rate constants relative to the autophosphorylation rate constant of CheA. Using the ratios of total protein concentrations, $\alpha_1 = [Y]_{\text{tot}}/[A]_{\text{tot}}$, $\alpha_2 = [B]_{\text{tot}}/[A]_{\text{tot}}$, and $\alpha_3 = [Z]_{\text{tot}}/[Y]_{\text{tot}}$, we obtain the transformed set of equations

$$\frac{d}{d\tau}[a_p] = A \cdot (1 - [a_p]) - \alpha_1\kappa_Y(1 - [y_p])[a_p] - \alpha_2\kappa_B(1 - [b_p])[a_p] \tag{9}$$

$$\frac{d}{d\tau}[y_p] = \kappa_Y(1 - [y_p])[a_p] - \kappa_1(\alpha_3 - [y_pz])[y_p] + \kappa_2[y_pz] \tag{10}$$

$$\frac{d}{d\tau}[y_pz] = \kappa_1(\alpha_3 - [y_pz])[y_p] - (\kappa_2 + \kappa_3)[y_pz] \tag{11}$$

$$\frac{d}{d\tau}[b_p] = \kappa_B(1 - [b_p])[a_p] - \kappa_{-B}[b_p]. \tag{12}$$

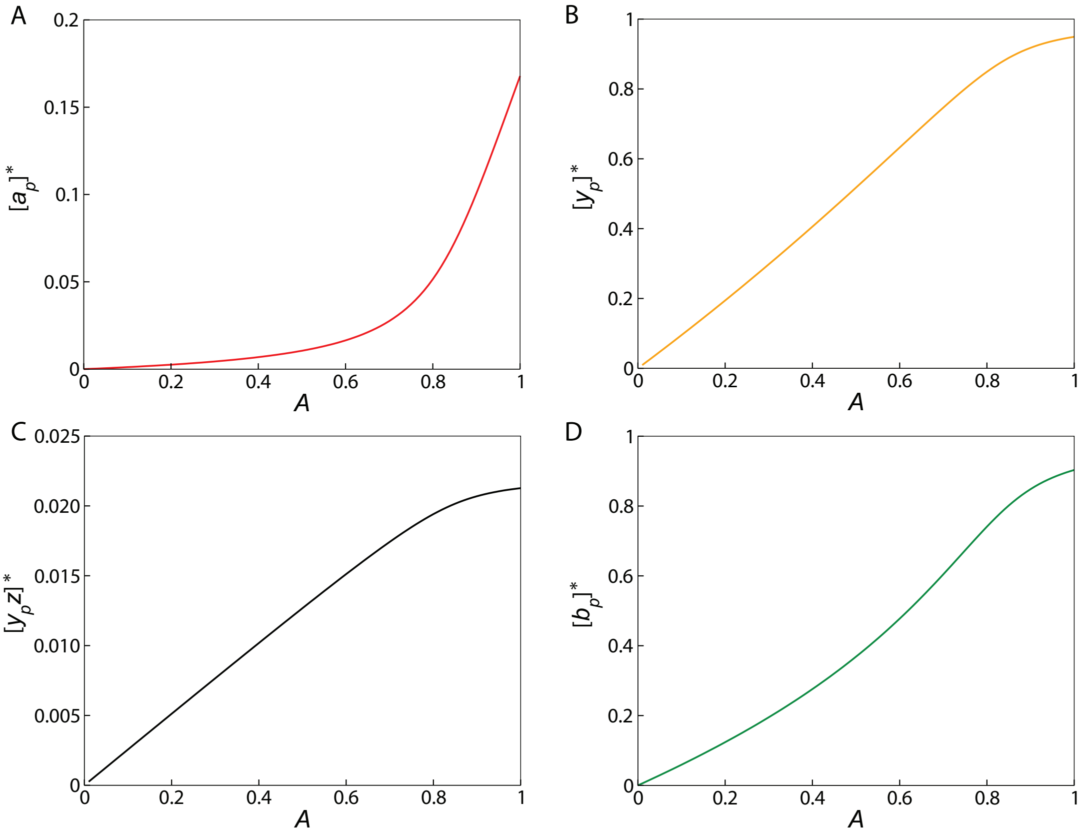

Figure 2: Steady-state concentrations of individual proteins and CheY-P/CheZ pairs for the whole-pathway model for Eq. 9-12, as a function of the receptor complex activity A. Note the different scales of the vertical axes. The adapted activity is $A^* \approx$1/3.

The transformed equation for the methylation level of receptors is obtained by replacing time $t \rightarrow \tau$, $g_R \rightarrow \gamma_R = g_R/k_A$ and $\hat{g}_B \rightarrow \gamma_B = \hat{g}_B B_{\mathrm{tot}}^2/k_A$ in Eq. 7, yielding

$$\frac{dm}{d\tau} = \gamma_R\,(1-A) - \gamma_B [b_p]^2 A. \tag{13}$$

The new parameter values of this transformed model are listed in Table 1.

2.2 Steady-state concentrations

We analyzed the steady-state concentrations of phosphorylated proteins and CheY-P/CheZ pairs. Setting the time-derivatives of Eq. 9-12 to zero, we solved for the steady-state concentrations of CheA-P, CheY-P and CheB-P, as well as the concentration of CheY-P/CheZ pairs as a function of the receptor complex activity A. The results are shown in Fig. 2. CheA-P shows a strong non-linear dependence on the activity A, i.e., it is strongly activated at high receptor complex activity. It is also notable that only a small fraction of the CheA concentration is phosphorylated at maximal receptor activity $A = 1$, which nicely fits estimates from *in vitro* measurements [7]. All other phosphorylated fractions of protein, as well as the concentration of CheY-P/CheZ pairs are approximately proportional to receptor complex activity A.

2.3 Time courses and steady-state assumption

We tested if the phosphorylation and CheY-P/CheZ association reactions, Eq. 9-12, are in quasi-steady state compared to the slower methylation and demethylation reactions of receptors, Eq. 13. For this purpose, we increased all rate constants for phosphorylation, dephosphorylation, as well as CheY-P/CheZ association and dissociation by one order of magnitude, such that concentrations

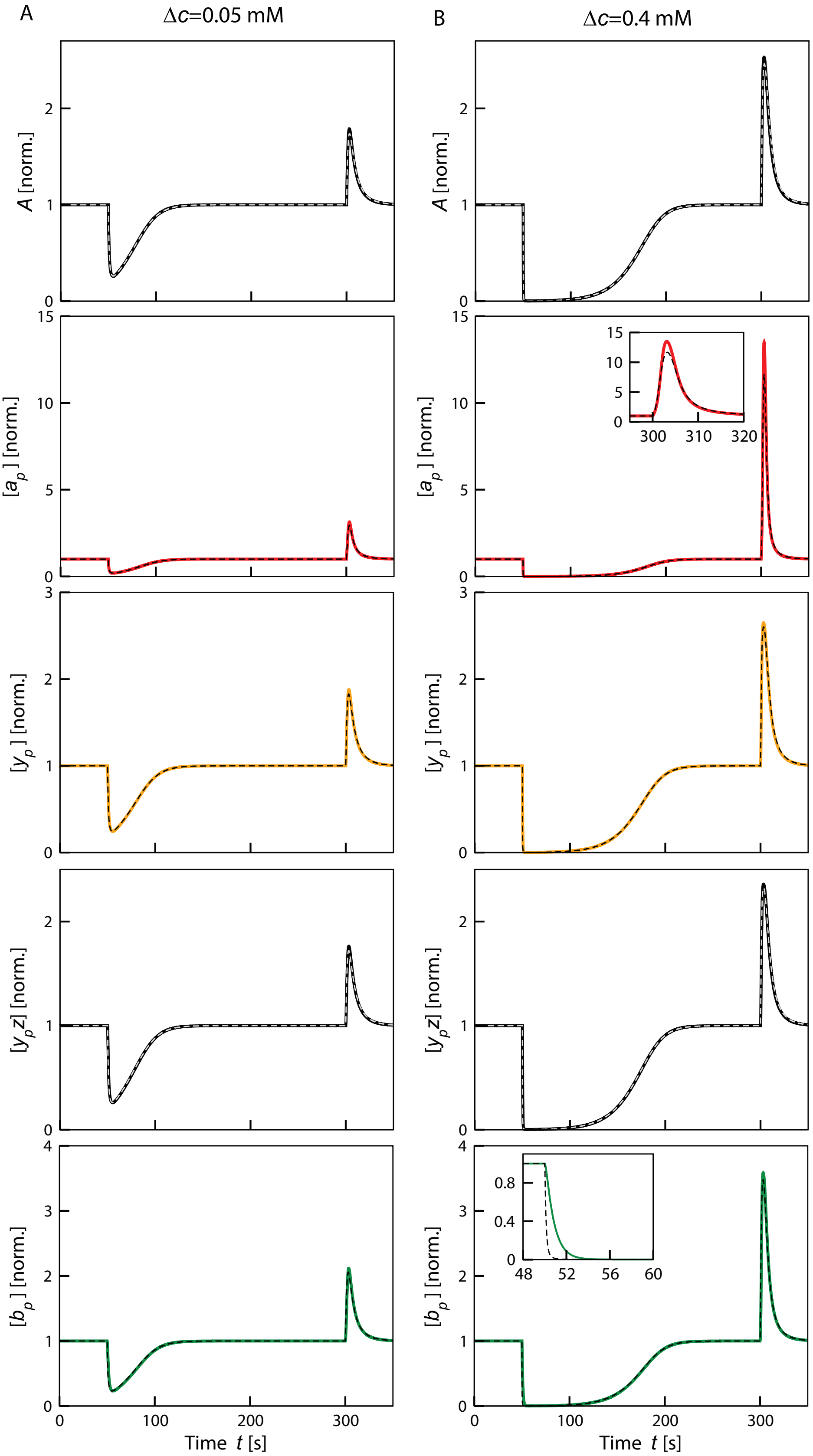

Figure 3: Time courses for the concentrations of phosphorylated proteins and CheY-P/CheZ pairs according to the whole-pathway model Eq. 9-13, using ambient MeAsp concentration c_0=0.1 mM and two different concentration step sizes, Δc=0.05 mM (column A) and Δc=0.4 mM (column B). Thick solid curves represent the model with parameter values as in table 1, thin dashed lines assume the quasi steady-state for all phosphorylation reactions (phosphorylation and dephosphorylation kinetic constants in the model increased by a factor 20). Insets zoom into the dynamics around the addition or removal time, respectively. Concentrations were normalized to their respective adapted values.

Table 1: Parameters of the whole-pathway model for chemotaxis signaling for Eq. 1-7, including references to literature values where possible, and rescaled parameters for Eq. 9-13. The literature values are given in parentheses where different from our parameter values. k_1 was determined by the condition that at steady-state with A^*=1/3, the concentration $[Y_p]^* = [Y]_{\rm tot}/3$ [8]. g_R was determined by the steady-state activity A^* and the value for $\hat{g}_B$.

Parameter	Value	Reference	Scaled parameter	Value
$[A]_{\rm tot}$	5 μM	[8]	α_1	1.94
$[B]_{\rm tot}$	0.28 μM	[9]	α_2	0.056
$[Y]_{\rm tot}$	9.7 μM	[9]	α_3	0.113
$[Z]_{\rm tot}$	1.1 μM	[8]	κ_Y	50
k_A	10 s^{-1}	[7]	κ_B	7.5
k_Y	100 μM^{-1} s^{-1}	[10]	κ_1	4.88
k_B	15 μM^{-1} s^{-1}	[10]	κ_2	0.05
k_1	5.0 μM^{-1} s^{-1}	[8]	κ_3	20
k_2	0.5 s^{-1}	[8]	κ_{-B}	0.135
k_3	200 s^{-1}	(30 s^{-1}) [8]	γ_R	0.0006
k_{-B}	1.35 s^{-1}	(0.35 s^{-1}) [11,12]	γ_B	0.0246
g_R	0.006 s^{-1}	—		
$\hat{g}_B$	3.14 μM^{-2} s^{-1}	—		

are forced to be in quasi steady-state at each time point. Comparing the results to the time courses with the original parameter values shown in Fig. 3, we found only minor deviations (exemplified in insets). Therefore, the above mentioned reactions are indeed in quasi-steady state to a good approximation. This, together with the approximate linearity of the steady-state concentration of CheY-P/CheZ pairs as function of receptor complex activity A, permits us to replace the number of FRET (CheY-P/CheZ) pairs by the receptor complex activity (with appropriate proportionality factors) as assumed in the main text. Similarly, Eq. 2 in the main text arises by replacing CheB-P concentration in the demethylation rate in Eq. 13 by the receptor complex activity A, where the methylation and demethylation rate constants are $g_R = \gamma_R k_A$ and $g_B = \gamma_B k_A \left([b_p]/A\right)^2 \approx \gamma_B k_A$, respectively.

3 Additional data and best fit of dynamic MWC model

In Fig. 4 we show additional, previously unpublished dose-response data measured as described in the main text (cf. Fig. 1 in the main text). The model in panel A is the dynamic MWC model from the main text. Panel B shows the best fit of the dynamic MWC model, where we used the demethylation rate constant g_B, the coefficients determining the receptor complex size as a linear function of ambient concentration, and the ligand dissociation constants of Tar and Tsr as fitting parameters. We found that parameters overall stay similar to the previously used parameters; in particular the ligand dissociation constants do not change significantly. The main difference is larger receptor complex sizes than determined by fitting the static MWC model to individual addition dose-response curves. To compensate for the larger complex sizes, the adaptation rates are also slightly increased, marking the trade-off between increased activity responses by larger complex sizes and reduced activity responses by faster adaptation (controlling for MeAsp concentration dynamics).

Figure 5 quantifies the difference between measured dose-response curves and the static, as well as the dynamic MWC model, respectively, as detailed in the main text (cf. Fig. 1 in main text). We plot squared errors for each addition and removal dose-response curve. While the error for the dynamic MWC model is slightly larger for addition curves, its error for removal curves is much

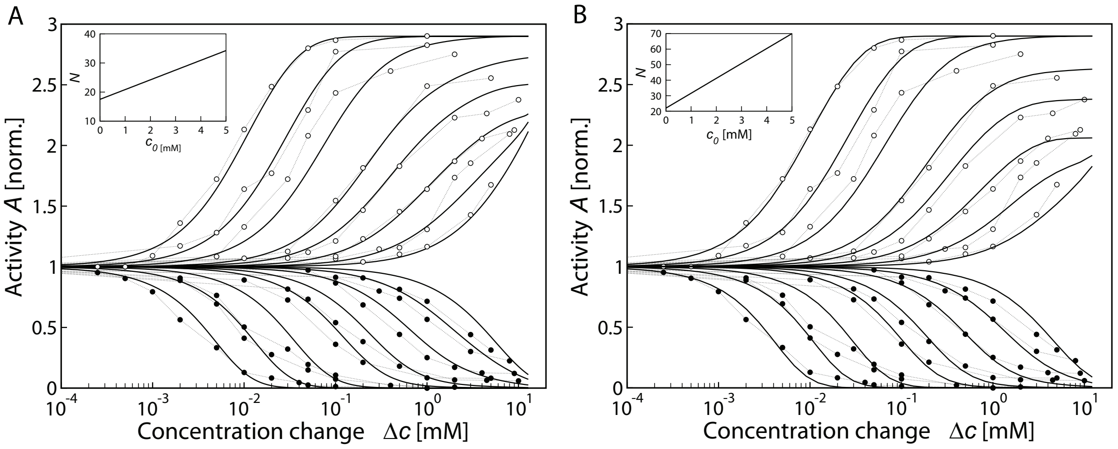

Figure 4: Dynamic MWC model. (A) Same as Fig. 1 in the main text (main panel), however showing additional, previously unpublished data. Shown are dose-response curves for wild-type cells to step changes of MeAsp concentration (after adaptation to ambient concentrations 0, 0.03, 0.1, 0.3, 0.5, 1, 2 and 5 mM). Symbols represent averaged response from FRET data as measured by Sourjik and Berg [6]. Filled and open circles correspond to response to addition and removal of attractant, respectively. Solid lines represent the dynamic MWC model of mixed Tar/Tsr-receptor complexes, including ligand rise (addition) and fall (removal), as well as adaptation (receptor methylation) dynamics. (B) Best global fit of dynamic MWC model with fitting parameters g_B=0.127 s^{-1}, K_a^{off}=0.02 mM, K_a^{on}=0.50 mM, K_s^{off}=216 mM, K_s^{on}=10^6 mM, as well as $a_0 = 22$ and a_1=9.6 mM^{-1} for the total receptor complex size $N = a_0 + a_1 c_0$.

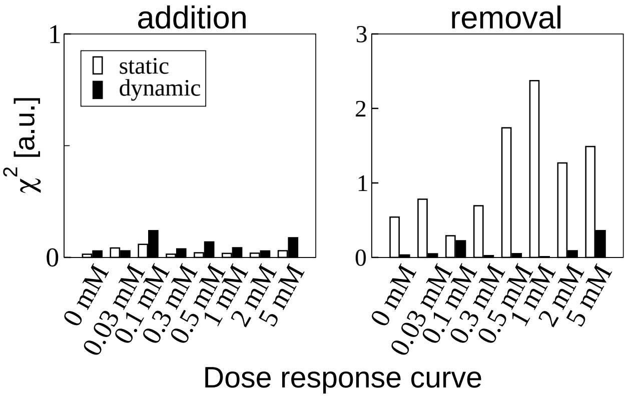

Figure 5: Residual absolute squared errors per addition (left panel) and removal (right panel) dose-response curve for the static and dynamic MWC model as shown in Fig. 1 in the main text. Note the different axis scales for addition and removal plots.

smaller than that for the static MWC model. Hence, the dynamic MWC model is suited better to describe the experimental data.

Figure 6 shows the free-energy change associated with each concentration step change. For increasing ambient concentrations, the free-energy changes generally decrease at a fixed concentration step change Δc. This is the reason for the reduced response amplitudes in the dynamic MWC model at large MeAsp step removals, because adaptation compensates for smaller free-energy changes at increasing ambient concentrations.

4 Parameters for static and dynamic MWC model

In Tab. 2, we list all parameters of the static and dynamic MWC model used for Fig. 1-3 in the main text and Fig. 4A in the Supplementary Text S1, as well as Fig. 4B and 8A in Text S1.

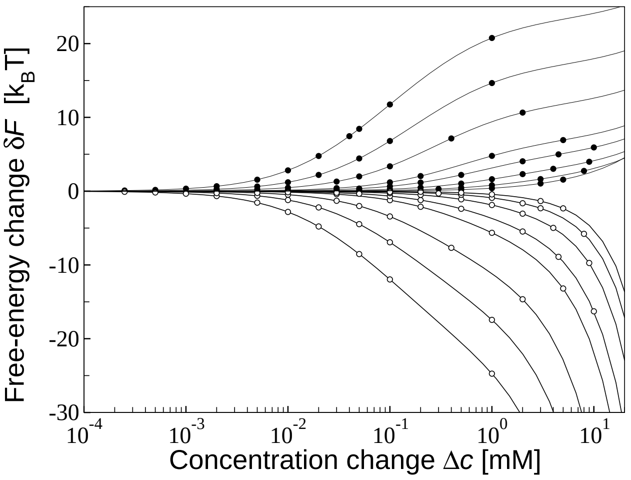

Figure 6: Changes in the free-energy difference $\delta F = F - F^*$ of a mixed-receptor complex upon concentration step changes Δc of MeAsp (lines), where F^* is the adapted free-energy difference. The curves correspond to ambient concentrations c_0=0, 0.03, 0.1, 0.3, 0.5, 1, 2 and 5 mM with free-energy differences for experimental concentration step changes indicated by symbols (filled circles for addition, open circles for removal of MeAsp).

Table 2: Fitting parameters for the static and dynamic MWC model. Parameters include dissociation constants for Tar and Tsr receptors in the *on* and *off* states, respectively, $K_a^{\rm off}$, $K_a^{\rm on}$, $K_s^{\rm off}$, $K_s^{\rm on}$ [13], the parameters of the linear approximation of the dependence of the receptor complex size on ambient concentration, $N(c_0) = a_0 + a_1 c_0$, as well as methylation and demethylation constants, g_R and g_B in Eq. 2 in the main text, respectively. Fitted parameters are indicated by crosses.

Parameter	Fig. 1-3 (main text), 4A (Text S1)				Fig. 4B (Text S1)		Fig. 8A (Text S1)	
	static		dynamic		dynamic (best fit)		static (best fit)	
$K_a^{\rm off}$ [mM]	0.02		0.02		0.02	x	0.056	x
$K_a^{\rm on}$ [mM]	0.5		0.5		0.50	x	0.15	x
$K_s^{\rm off}$ [mM]	100		100		216	x	100	
$K_s^{\rm on}$ [mM]	10^6		10^6		10^6	x	10^6	
a_0	17.5	x	17.5		22	x	37	x
a_1 [mM^{-1}]	3.35	x	3.35		9.6	x	-0.78	x
g_R [s^{-1}]	N/A		0.0069		0.0079		N/A	
g_B [s^{-1}]	N/A		0.11	x	0.127	x	N/A	

5 Effect of receptor complex size on data collapse

We found from fitting the MWC model to dose-response curves from FRET that receptor complex size increases with ambient concentration (Fig. 2A in the main text). Hence, we would like to determine how the data collapse depends on this effect. According to Eq. 4 in the main text, the rate of activity change is proportional to the receptor complex size N. As we do not have a model which describes how receptor complex size changes in time in response to concentration changes, we plot in Fig. 7 the data collapse for different N corresponding to the concentrations used in the experiments. This provides the envelope in which the data collapse is expected to change with N. We find that the data collapse does not change very much compared to the data collapse for ambient concentration c_0, and hence we neglected the effect of changing complex size in Fig. 3 in the main text.

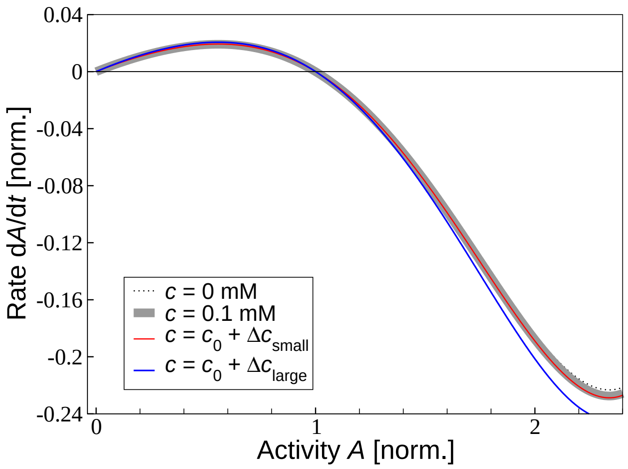

Figure 7: Effect of ligand concentration-dependent receptor complex size $N(c)$ on the predicted data collapse according to Eq. 4 in the main text. Plotted are the predicted functions $f(A)$ for N corresponding to ambient concentration $c = 0.1$ mM (thick gray line), zero ambient (buffer; black dotted line), and concentration upon small (red line) and large (blue line) concentration changes as in Fig. 3 in the main text.

6 Unsuitable receptor signaling models

We tested four alternative models for receptor signaling in an attempt to find a model, which describes the gradually reduced response amplitudes upon MeAsp removals at increasing ambient concentrations (cf. Fig. 1 in main text) without relying on adaptation and MeAsp dynamics. Dose-response curves for each model are shown in Fig. 8. We found none of the models produced a satisfying fit to the experimental data.

A Saturation model

While ligand dissociation constants for the Tar receptor were previously determined from FRET data [13], slightly different values may lead to saturation of Tar receptors at smaller concentrations of MeAsp and reduced response amplitudes. Figure 8A shows a fit of the static MWC model to addition as well as removal data. We fitted the parameters of the linear relationship between the receptor complex size and ambient concentration c_0, as well as the ligand dissociation constants for the Tar receptor, K_a^{on} and K_a^{off}. We find an unsatisfying fit, especially for the response to addition of MeAsp. Furthermore, the determined receptor complex size decreases with ambient concentration (see *Inset*). This contradicts experiments which indicate an increasing receptor complex size [14], as well as stabilization of polar receptor clusters with increasing receptor methylation level (corresponding to increasing ambient concentration) [15].

B Imprecise adaptation model

Figure 8B shows the effect of imprecise adaptation on the response amplitudes. For simplicity, we assume a linear decline of the adapted activity $A^*(c_0)$ with increasing ambient concentration c_0, with $A^*(0) = 1/3$ and a 20 percent imprecision at concentration 10 mM (see *Inset*). We observe that imprecise adaptation has only a small effect on the response amplitudes. Furthermore, imprecise adaptation tends to increase response amplitudes at high ambient concentrations due to normalization by a decreasing value of $A^*(c_0)$.

C Phase-separation model

In this model, a fraction w of mixed receptor complexes composed of Tar and Tsr receptors form homogeneous receptor complexes of only Tar and only Tsr receptors at high ambient concentrations. This separation reduces activity amplitudes at concentrations below the ligand dissociation constant

$K_s^{\rm off}$ for Tsr, as complexes of Tsr do not contribute to the response. The total activity from mixed and homogeneous receptor complexes is

$$A = [1 - w(c_0)]\, A^{\rm mixed} + w(c_0)\left(\nu_a A^{\rm Tar} + \nu_s A^{\rm Tsr}\right). \tag{14}$$

The individual activities of mixed, $A^{\rm mixed}$, and homogeneous receptor complexes of Tar, $A^{\rm Tar}$, and Tsr, $A^{\rm Tsr}$, were calculated according to the static MWC model. Mixed receptor complexes are composed of Tar and Tsr with ratio $\nu_a : \nu_s$=1:1.4. Homogeneous receptor complexes of Tar and Tsr, respectively, have the same ratio. The resulting dose-response curves for this model are shown in Fig. 8C, assuming the probability of separation $w(c_0)$ from the *Inset*. As the ambient concentration does not correspond nicely to the data points with decreasing response, we did not find a well-fitting function $w(c_0)$. Furthermore, this model predicts a smaller response to MeAsp when cells are pre-adapted to a ligand for which Tsr, but not Tar is sensitive (e.g. Serine). This contradicts experiments, which show that cells remain sensitive (Ref. [16] and V.S., manuscript in preparation).

D Receptor lattice model

In the static MWC model the absolute cooperativity of the receptors in a complex results in a saturating response upon removal of attractant (cf. *Inset* of Fig. 1 in main text). Here, we consider an Ising lattice of N_T two-state receptor trimers, where each trimer is coupled to neighboring trimers with finite interaction strength. We ask if a weaker coupling between receptors can describe the dose-response data, and in particular the reduced response amplitudes for removals.

Figure 8D shows dose-response curves for different interaction strengths. We find, that in order to describe the addition data, a strong interaction between neighboring trimers has to be assumed. In this limit, the lattice model resembles the MWC model where all lattice sites are infinitely strongly coupled with all other receptors. In the *Inset* of Fig. 8D we show the activity distribution from all lattice states. As expected, the distribution becomes increasingly bimodal around the two states with *all receptors on* and *all receptors off*.

In the following we describe the details of the model and our simulations. We used a 4-by-4 square lattice of mixed receptor trimers with periodic boundary conditions. Each trimer consisted of Tar and Tsr receptors with probabilities ν_a and ν_s, respectively, where ν_a:ν_s=1:1.4 is the *in vivo* ratio of Tar and Tsr in a cell. The distribution of Tar and Tsr in trimers on the lattice was the same in all simulations. Furthermore, each trimer has only two states, *on* and *off*. We numerate all possible states of the whole lattice (in total $n = 2^{16}$ states for a 4-by-4 lattice, i.e. N_T=16 receptor trimers). Assuming the lattice is in equilibrium, we can calculate the distribution of individual lattice states, and hence the average activity of the lattice. The probability of each lattice state depends on its energy, which has a contribution from the free-energy difference between the *on* and *off* states of each trimer and from the interaction between neighboring trimers. The free-energy difference of trimer j is computed according to the MWC model

$$F^j = \epsilon(m^j) + \sum_{l=1}^{3} \ln\left(\frac{1 + c/K_l^{\rm off}}{1 + c/K_l^{\rm on}}\right), \tag{15}$$

where the index $l = a, s$ describes the receptor type, Tar or Tsr, within a trimer. The average methylation level of receptors in a trimer j is denoted by m^j. The methylation energy is $\epsilon(m^j) = 3 \cdot (1 - 0.5\, m^j)$.

The interaction energy between neighboring trimers depends on their respective states. If they are in the same state (both *on* or both *off*), we assign the interaction energy J, if they are in different states, we assign the interaction energy $-J$. The total energy E_k of a lattice state k is determined by summing over all free-energy differences of individual trimers and interaction energies between neighboring trimers.

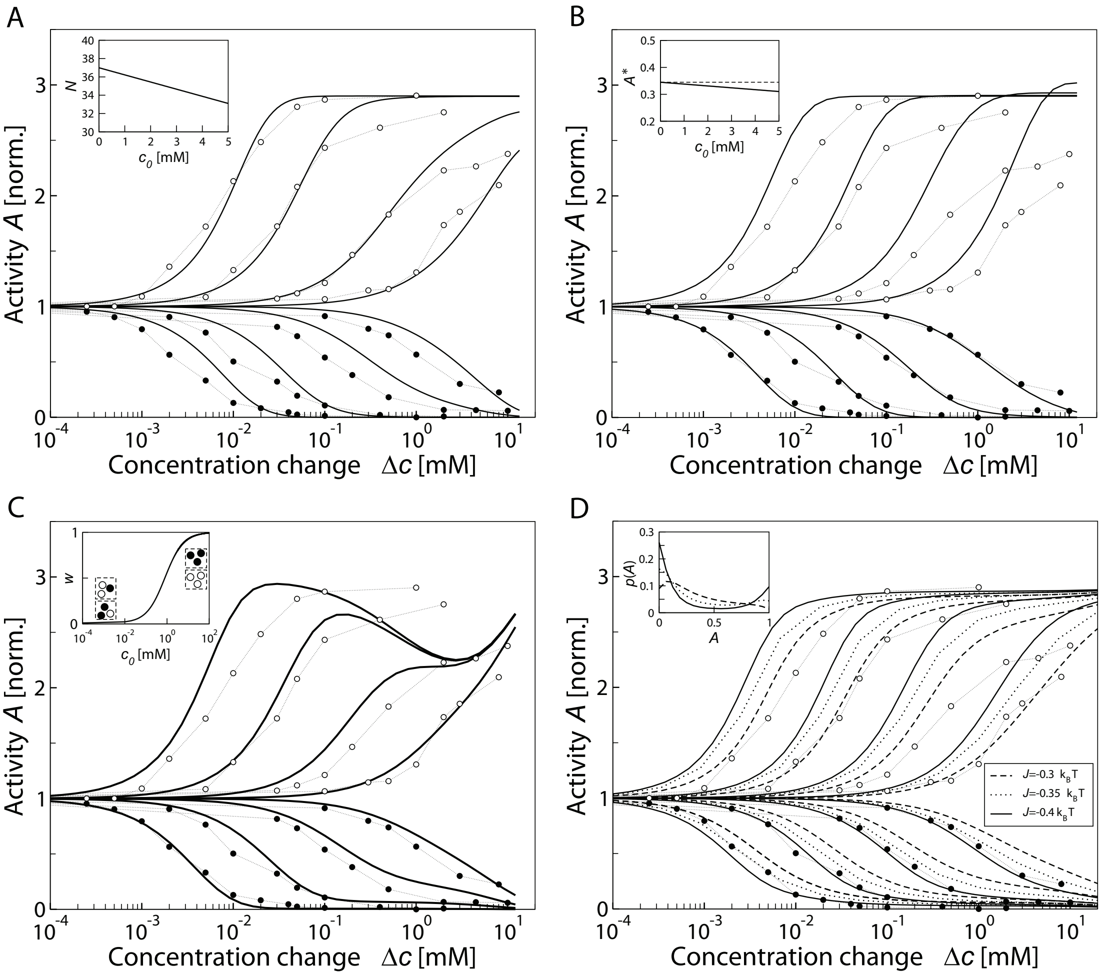

Figure 8: Alternative models for receptor signaling. (A) Saturation model. MWC model with optimized dissociation constants $K_a^{\text{off}} = 0.056$ mM and $K_a^{\text{on}} = 0.15$ mM. (A *Inset*) Total receptor complex size $N = a_0 + a_1 c_0$ for fitted parameters a_0=37 and a_1=-0.78 mM^{-1}. (B) Imprecise adaptation model. (B *Inset*) The steady-state activity decreases with ambient concentration, $A^*(c_0)/A^*(0) = 1 - (0.2/10 \text{ mM})c_0$, where 20% imprecision is reached at 10 mM. (C) Phase-separation model. Receptor complexes are found in separated complexes with probability w depending on the ambient concentration c_0. Receptor complex size is assumed constant, N =18, for mixed and homogeneous receptor complexes. (C *Inset*) The probability $w(c_0) = p_1 + p_2 \frac{c_0}{c_0 + p_3}$, with p_1=0.01, p_2=0.99, p_3=0.8 mM. (D) Receptor lattice model. Mixed trimers of Tar and Tsr dimers are arranged on a 4×4 square lattice with periodic boundary conditions. The average activity of the lattice was calculated by exact enumeration. An attractive interaction between neighboring trimers in the same state was assumed, with interaction energy J=-0.4 k_BT (solid line), J=-0.35 k_BT (dotted line), and J=-0.3 k_BT (dashed line). (D *Inset*) Corresponding distributions of activities from all states (lattice configurations) when adapted to zero ambient concentration.

The methylation level m^j of each trimer j cannot be calculated analytically due to the finite coupling strength between receptor trimers, and hence was determined numerically using our adaptation model

$$\frac{dm^j}{dt} = g_R(1 - A^j) - g_B(A^j)^3. \quad (16)$$

According to this model, the methylation level m^j of the trimer j depends on its average activity

$$A^j = \frac{1}{Z}\sum_{k=1}^{n} s_k^j e^{-E_k}, \quad (17)$$

where $Z = \sum_k e^{-E_k}$ is the partition function, i.e. the sum over all lattice states and s_k^j is the state (1=*on*, 0=*off*) of trimer j in lattice state k. The steady-state of Eq. 16 determines the methylation level of each trimer, and therefore the adapted free-energy difference $\epsilon(m^j)$ in Eq. 15. The average activity of the whole lattice is determined by calculating the average trimer activity

$$A = \frac{1}{N_T}\sum_{j=1}^{N_T} A^j, \quad (18)$$

where N_T is the number of trimers on the lattice.

7 Comparison of different adaptation models

In Fig. 4 in the main text we compare different adaptation models to FRET data by collapsing the time courses, plotting the rate of activity change dA/dt as a function of the activity A. Here, we describe in detail the different models analyzed. In all of the models we assume precise adaptation, i.e. that methylation and demethylation rates only depend on the receptor complex activity. For each adaptation model, we use a least-squares fit to the FRET data to determine the methylation and demethylation rate constants, assuming an adapted activity A^*=1/3 and receptor complex size $N = 17.8$. The parameters and quality of fit χ^2 for each of the models are listed in Tab. 3.

Our model Eq. 2 in the main text

$$\frac{dm}{dt} = g_R(1 - A) - g_B A^3 \quad (19)$$

is denoted by "$(1 - A)$, A^3", referring to the activity dependence of the methylation and demethylation rates, respectively. The best fit to the rate of activity change from FRET (fitting parameter g_R=0.0019 s^{-1}, resulting in g_B=0.030 s^{-1} and quality of fit χ^2=0.0021), and a representative time course for this model are shown in Fig. 4A and B in the main text, respectively (red solid lines). Note that this model describes the experimental data well, even at high activities. This model also shows a strong asymmetry in the time course with slow adaptation to addition and rapid adaptation to removal of MeAsp (cf. Fig. 2C in the main text).

We considered a variation of this model, denoted by "$(1 - A)$, A^2", without cooperativity of CheB-P molecules,

$$\frac{dm}{dt} = g_R(1 - A) - g_B A^2, \quad (20)$$

where only one CheB-P molecule is necessary for demethylation of a receptor. Together with one factor A from the activity of receptors, this leads to a demethylation rate proportional to A^2. While this model is almost as well-suited to describe the rate of activity change from FRET as our main model (fitting parameter g_R=0.0031 s^{-1}; g_B=0.017 s^{-1}, χ^2=0.0022; see Fig. 4A in main text), the asymmetry of adaptation to addition and removal of MeAsp is less pronounced (Fig. 4B in main text). Fitting dose-response data using this adaptation model resulted in adaptation rates which were much higher than observed in FRET time courses.

Table 3: Parameters of the adaptation models when fitted to the rate of activity change from FRET shown in Fig. 4A in the main text. The size of receptor complexes was assumed to be $N = 17.8$ in all models. [a] $K_1 = K_r/[T]$ and $K_2 = K_b/[T]$, where K_r=0.39 μM and K_b=0.54 μM are taken from Ref. [17]. [b] $K_2 = K_b/[T]$ with K_b=1.25 μM [18]. The concentration of receptors is $[T]$=17 μM.

Adaptation model	g_R (fitted)	other parameters	χ^2
"$(1-A)$, A^3"	0.0019 s^{-1}	g_B=0.030 s^{-1}	0.0021
"$(1-A)$, A^2"	0.0031 s^{-1}	g_B=0.017 s^{-1}	0.0022
"$\frac{1-A}{1-A+K_1}$, $\frac{A}{A+K_2}$"	0.0188 s^{-1}	g_B=0.020 s^{-1}<br>K_1= 0.0229[a]<br>K_2= 0.0318[a]	0.0036
"$\frac{1-A}{1-A+K_1}$, $\frac{A^2}{A+K_2}$"	0.0046 s^{-1}	g_B=0.014 s^{-1}<br>K_1= 0.0229[a]<br>K_2= 0.0318[a]	0.0025
"const, $\frac{A}{A+K_2}$"	0.00318 s^{-1}	g_B=0.014 s^{-1}<br>K_2= 0.0735[b]	0.0032

Furthermore, the model denoted by "$(1-A)$, A" without CheB-P feedback [19–22]

$$\frac{dm}{dt} = g_R(1-A) - g_B A \tag{21}$$

yields the fitting parameter g_R=0.0048 s^{-1}, resulting in g_B=0.0091 s^{-1} and quality of fit χ^2=0.0025. Both, the fit of this model to the rate of activity change from FRET, and time courses, are described worse than with the other two models.

Another class of adaptation models was proposed in Ref. [17] where the idea of ultrasensitivity to the adaptation dynamics of CheR and CheB-P was introduced. This model was proposed mainly for small changes in activity, such as for fluctuations around the steady-state activity due to noise. We denote by "$(1-A)/(1-A+K_1)$, $A/(A+K_2)$" the following model

$$\frac{dm}{dt} = g_R\frac{1-A}{1-A+K_1} - g_B\frac{A}{A+K_2}. \tag{22}$$

In this model, CheR (CheB) methylates (demethylates) inactive (active) receptors with Michaelis-Menten-type kinetics with Michaelis-Menten constant K_1 (K_2). In this model, there is no CheB-P feedback on the demethylation rate. If K_1 and K_2 are small, the adaptation rate depends only weakly on the receptor activity. This results in long adaptation (relaxation) times, as well as strong sensitivity to protein fluctuations of either CheR or CheB through rates g_R and g_B. We used $K_1 = K_r/[T] = 0.0229$ and $K_2 = K_b/[T] = 0.0318$, where we took K_r and K_b from [17] and the concentration of receptors is $[T]$=17 μM. As shown in Fig. 4A in the main text, the model without CheB-P feedback "$(1-A)/(1-A+K_1)$, $A/(A+K_2)$" does not describe the rate of activity change from FRET (fitting parameter g_R=0.0188 s^{-1}; g_B=0.020 s^{-1}, χ^2=0.0036). Furthermore, the time course shown in panel B looks qualitatively different from experimental time courses (cf. Fig. 2C in main text).

A variant of the model also includes CheB-P feedback, which introduces another factor A in the demethylation rate [17]. We denote this model by "$(1-A)/(1-A+K_1)$, $A^2/(A+K_2)$", which corresponds to

$$\frac{dm}{dt} = g_R\frac{1-A}{1-A+K_1} - g_B\frac{A^2}{A+K_2}. \tag{23}$$

This model fits the FRET activity change in Fig. 4A in the main text relatively well (fitting parameter g_R=0.0046 s^{-1}; g_B=0.014 s^{-1}, χ^2=0.0025). However, this model is not very different from the simpler model "$(1-A)$, A^2", as the CheB-P feedback introduces a strong activity-dependence.

In the model suggested by Barkai and Leibler [18] CheR methylation does not depend on the activity state of receptors, and hence active, as well as inactive receptors get methylated. The kinetics of the methylation level is described by

$$\frac{dm}{dt} = g_R - g_B \frac{A}{A + K_2}, \tag{24}$$

where the parameter value $K_2 = K_b/[T]$=0.074 with K_b=1.25 μM [18], and $[T]$ as above. Note that this model is a special case of above model "$(1-A)/(1-A+K_1)$, $A/(A+K_2)$" with K_1=0. Fitting to the FRET activity change yields g_R=0.00318 s^{-1}, resulting in g_B=0.014 s^{-1} and quality of fit χ^2=0.0032. The predicted data collapse, as well as time courses are very similar to the model "$(1-A)/(1-A+K_1)$, $A/(A+K_2)$", and is therefore not plotted in Fig. 4 in the main text.

8 Analysis of adaptation noise

The receptor methylation level is subject to fluctuations due to the random nature of methylation and demethylation events. However, the adaptation dynamics also filters fluctuations in ligand concentration (translated into fluctuations of the receptor activity), averaging over and smoothing high-frequency noise by its slower dynamics. Here, we estimate the variance of the methylation level of a receptor complex due to these two noise sources. Equation 2 of the main text describes the deterministic kinetics of the average methylation level of receptors in a mixed receptor complex,

$$\frac{dm}{dt} = g_R(1-A) - g_B A^3. \tag{25}$$

Now, we consider the kinetics of the total methylation level of a receptor complex. The total methylation level M is the sum of the individual methylation levels m_i of all receptors in a complex, $M = \sum_{i=1}^{N} m_i$, with N the number of receptors per complex. The rate of change of the total methylation level is

$$\frac{dM}{dt} = N_R k_R (1-A) - N_B k_B A^3, \tag{26}$$

where we explicitly indicated the number of the modifying CheR and CheB-P molecules, N_R and N_B, respectively. The modification rates for a single receptor are related to the rates for a receptor complex via $\tilde{g}_R = N_R k_R/N$ and $g_B = N_B k_B/N$, respectively. To describe fluctuations about the mean total methylation level due to methylation and demethylation events, we introduce the noise $\eta(t)$ and write

$$\frac{dM}{dt} = N_R k_R (1-A) - N_B k_B A^3 + \eta(t). \tag{27}$$

We assume $\eta(t)$ is the sum of individual noise terms contributed from each modifying enzyme CheR and CheB-P acting on groups of receptors, so-called *assistance neighborhoods* [19, 20, 23],

$$\eta(t) = \sum_{i=1}^{N_R} \eta_{R(i)}(t) + \sum_{i=1}^{N_B} \eta_{B(i)}(t), \tag{28}$$

where $\eta_{R(i)}$ and $\eta_{B(i)}$ are independent Gaussian white noises with zero mean $\langle \eta_{R(i)}(t) \rangle = \langle \eta_{B(i)}(t) \rangle$=0, autocorrelations $\langle \eta_{R(i)}(t) \eta_{R(i)}(t') \rangle = q_R \cdot \delta(t-t')$ and $\langle \eta_{B(i)}(t) \eta_{B(i)}(t') \rangle = q_B \cdot \delta(t-t')$, and vanishing cross-correlations. To estimate the noise intensities q_R and q_B, we assume that the number of methyl groups, which are added (removed) by each enzyme molecule CheR (CheB-P) in a time interval, are Poisson distributed, i.e. their variance equals the mean number of added (removed) methyl groups. Therefore, the noise intensity q_R associated with each CheR molecule is determined by its mean rate of methylation,

$$q_R = k_R(1-A^*). \tag{29}$$

Similarly, the noise intensity q_B for demethylation is

$$q_B = k_B A^{*3}, \tag{30}$$

where we only consider noise from one molecule of CheB-P. We are interested in the steady-state fluctuations of the total methylation level. Therefore, we linearize Eq. 26 around the steady state to obtain the kinetics of the deviation δM from the mean methylation level

$$\frac{d(\delta M)}{dt} = -\left(N_R k_R + 3N_B k_B A^{*2}\right)\delta A + \eta(t) \tag{31}$$

$$= -\left(N_R k_R + 3N_B k_B A^{*2}\right)\left(\frac{\partial A}{\partial F}\right)\left(\frac{\partial F}{\partial M}\cdot\delta M + \frac{\partial F}{\partial c}\cdot\delta c\right) + \eta(t). \tag{32}$$

In the second step, we used that the receptor complex activity is subject to fluctuations from the methylation level, as well as the ligand concentration. The derivative of receptor complex activity with respect to the free-energy difference (at steady state) is given by

$$\frac{\partial A}{\partial F} = -A^*(1-A^*). \tag{33}$$

The total methylation level of a receptor complex enters the free-energy difference through

$$F = \underbrace{N - \frac{1}{2}M}_{=\sum_{i=1}^{N}\left(1-\frac{1}{2}m_i\right)} + \nu_a N \ln\left(\frac{1+c/K_a^{\text{off}}}{1+c/K_a^{\text{on}}}\right) + \nu_s N \ln\left(\frac{1+c/K_s^{\text{off}}}{1+c/K_s^{\text{on}}}\right), \tag{34}$$

where m_i are the methylation levels of receptors i. Therefore, the derivative of the free-energy difference F with respect to M is given by

$$\frac{\partial F}{\partial M} = -\frac{1}{2}. \tag{35}$$

The derivative of the free-energy difference F with respect to c is given by

$$\frac{\partial F}{\partial c} = \nu_a N\left(\frac{1}{c+K_a^{\text{off}}} - \frac{1}{c+K_a^{\text{on}}}\right) + \nu_s N\left(\frac{1}{c+K_s^{\text{off}}} - \frac{1}{c+K_s^{\text{on}}}\right) \equiv \mu. \tag{36}$$

In summary, the kinetics of δM is determined by

$$\frac{d(\delta M)}{dt} = -\underbrace{\left(N_R k_R + 3N_B k_B A^{*2}\right)A^*(1-A^*)}_{\equiv\lambda}\cdot\left(\frac{1}{2}\delta M - \mu\delta c\right) + \eta(t). \tag{37}$$

To calculate the variance of the methylation level, we Fourier-transform Eq. 37,

$$i\omega\delta\hat{M} = -\lambda\left(\frac{1}{2}\delta\hat{M} - \mu\delta\hat{c}\right) + \hat{\eta}, \tag{38}$$

where the hat symbol denotes the Fourier transform. The power spectrum S_M of fluctuations in M is defined as the average of the absolute value squared of $\delta\hat{M}$

$$S_M(\omega) = \langle|\delta\hat{M}|^2\rangle = \frac{q_M + \lambda^2\mu^2\langle|\delta c|^2\rangle}{\omega^2 + \lambda^2/4}. \tag{39}$$

Here, q_M denotes the noise intensity of methylation and demethylation, and $\lambda^2\mu^2\langle|\delta c|^2\rangle$ is due to the uncertainty from the ligand concentration[1], where we assumed the two contributions are independent. In this formula, we see explicitly the noise filtering of fluctuations in ligand concentration by the kinetics of the methylation level, given by the frequency-dependent factor.

[1]Fluctuations of the ligand concentration characterized by $\langle\delta c^2\rangle$ can be quantified as presented in Ref. [24,25] by

$$\langle\delta c^2\rangle = \frac{\alpha}{\pi a D\tau}\cdot c, \tag{40}$$

which corresponds to the time-averaged low-frequency limit of the noise power spectrum [25,26]. The parameter a is the size of the ligand binding site of a receptor, D is the ligand diffusion constant, and τ is an averaging time due to slower downstream reactions. The parameter α is of the order one and depends on further receptor details [25,26]. Using $\alpha \approx 1$, a=1 nm, D=100 μm^2/s, a typical ligand concentration $c = \sqrt{K_a^{\text{off}}K_a^{\text{on}}} = 0.1$ mM [21], and $\tau = 1/k_A = 0.1$ s corresponding to slow autophosphorylation of CheA, we obtain $\langle\delta c^2\rangle = 5\cdot 10^{-6}$ mM^2.

In the following, we calculate the variance of the methylation level of a receptor complex only due to methylation and demethylation events. As $\eta(t)$ is composed of independent white noises, its total noise intensity q_M is the sum of the individual noise intensities,

$$q_M = \langle |\hat{\eta}|^2 \rangle = N_R q_R + N_B q_B = 2 N_R q_R = 2 N_R k_R (1 - A^*). \tag{41}$$

The last equality uses the fact that at steady state methylation and demethylation rates balance each other in Eq. 26. To calculate the variance of the methylation level we need to integrate the power spectrum over all frequencies ω,

$$\langle \delta M^2 \rangle = \int \frac{d\omega}{2\pi} \frac{q_M}{\omega^2 + \lambda^2/4} = \frac{2 q_M}{\lambda}, \tag{42}$$

and obtain

$$\begin{aligned} \langle \delta M^2 \rangle &= \frac{2 N_R q_R}{\left(N_R k_R + 3 N_B k_B A^{*2}\right) A^* (1 - A^*)} = \frac{2 g_R}{\left(g_R + 3 g_B A^{*2}\right) A^*} = \frac{2}{A^* + 3(1 - A^*)} \\ &= 0.87. \end{aligned} \tag{43}$$

Here, we used that the adapted activity is $A^* \approx 1/3$, and that the relation between the methylation and demethylation rate constants g_R and g_B is given by the steady state of the methylation kinetics Eq. 25,

$$g_B = g_R \frac{1 - A^*}{A^{*3}}. \tag{44}$$

This result can be compared to results for other adaptation models previously reported in the literature. Reference [20] uses a linear dependence of methylation and demethylation rates on the receptor activity, instead of the nonlinear dependence in Eq. 25,

$$\frac{dm}{dt} = g_R (1 - A) - g_B A. \tag{45}$$

In an equivalent approach using assistance neighborhoods as described above, the authors calculate the variance of the total methylation level to be

$$\langle \delta M^2 \rangle = \frac{1}{|\partial F / \partial M|} = 2. \tag{46}$$

Hence, the variance of the total methylation level of a receptor complex is reduced for adaptation kinetics with strong activity dependence of the demethylation rate (Eq. 25), compared to the linear adaptation model (Eq. 45). The reason for this is the stronger negative feedback, leading to the rapid attenuation of fluctuations in the receptor complex activity. Mathematically, the prefactor of the linearized demethylation rate in Eq. 43 leads to the reduction of the variance of the methylation level of the receptor complex.

9 Quantification of adaptation imprecision

In Fig. 9 we quantify the imprecision of adaptation. Cells were adapted to 100 μM ambient concentration with adapted pre-stimulus activity A^*_{pre} measured by FRET. Concentration step changes of various sizes were added, and cells adapted to the new concentration with post-stimulus adapted activity A^*_{post}. We define a measure of imprecision as

$$\text{Imprecision} = \frac{A^*_{\text{post}} - A^*_{\text{pre}}}{A^*_{\text{pre}}}. \tag{47}$$

We find that adaptation is highly variable from experiment to experiment (high standard deviation). However, cells are found to consistently adapt imprecisely at high concentrations.

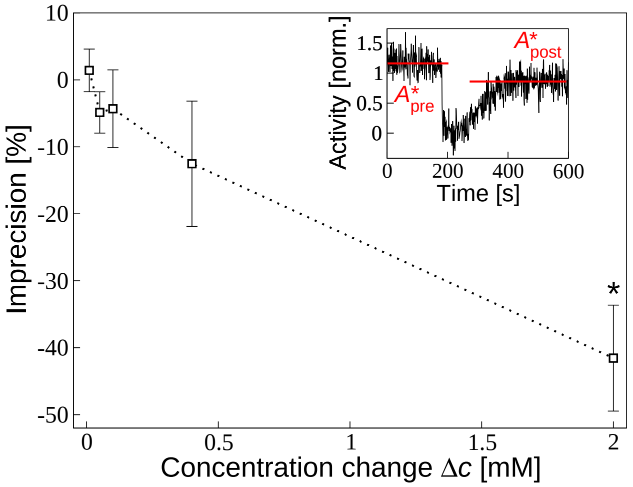

Figure 9: Imprecision of adaptation. FRET time courses were measured for cells adapted to 0.1 mM ambient concentration, and subject to various concentration step changes Δc. Levels of adapted FRET activity were determined before and after each added concentration step change, and the imprecision was calculated as $(A^*_{\rm post} - A^*_{\rm pre})/A^*_{\rm pre}$. Symbols correspond to mean values of imprecision, and error bars indicate the standard mean error based on three replicates. The star indicates statistically significant difference from zero with Student's t-test p-value smaller than 0.05. (*Inset*) Example FRET time course for Δc=2 mM with adapted pre- and post-stimulus activity indicated.

References

1. Berg HC (2000) Motile behavior of bacteria. Phys Today 53: 24-29.
2. Falke JJ, Hazelbauer GL (2001) Transmembrane signaling in bacterial chemoreceptors. Trends Biochem Sci 26: 257-265.
3. Sourjik V (2004) Receptor clustering and signal processing in *E. coli* chemotaxis. Trends Microbiol 12: 569-576.
4. Wadhams GH, Armitage JP (2004) Making sense of it all: bacterial chemotaxis. Nat Rev Mol Cell Biol 5: 1024-1037.
5. Kentner D, Sourjik V (2009) Dynamic map of protein interactions in the *Escherichia coli* chemotaxis pathway. Mol Syst Biol 5: 238.
6. Sourjik V, Berg HC (2002) Receptor sensitivity in bacterial chemotaxis. Proc Natl Acad Sci U S A 99: 123-127.
7. Wolanin PM, Baker MD, Francis NR, Thomas DR, DeRosier DJ, et al. (2006) Self-assembly of receptor/signaling complexes in bacterial chemotaxis. Proc Natl Acad Sci U S A 103: 14313-14318.
8. Sourjik V, Berg HC (2002) Binding of the *Escherichia coli* response regulator CheY to its target measured *in vivo* by fluorescence resonance energy transfer. Proc Natl Acad Sci U S A 99: 12669-12674.
9. Li M, Hazelbauer GL (2004) Cellular stoichiometry of the components of the chemotaxis signaling complex. J Bacteriol 186: 3687-3694.
10. Stewart RC, Jahreis K, Parkinson JS (2000) Rapid phosphotransfer to CheY from a CheA protein lacking the CheY-binding domain. Biochemistry 39: 13157-13165.
11. Bray D, Bourret RB (1995) Computer analysis of the binding reactions leading to a transmembrane receptor-linked multiprotein complex involved in bacterial chemotaxis. Mol Biol Cell 6: 1367-1380.

12. Stewart RC (1993) Activating and inhibitory mutations in the regulatory domains of the methylesterase in bacterial chemotaxis. J Biol Chem 268: 1921-1930.

13. Keymer JE, Endres RG, Skoge M, Meir Y, Wingreen NS (2006) Chemosensing in *Escherichia coli*: two regimes of two-state receptors. Proc Natl Acad Sci U S A 103: 1786-1791.

14. Endres RG, Oleksiuk O, Hansen CH, Meir Y, Sourjik V, et al. (2008) Variable sizes of *Escherichia coli* chemoreceptor signaling teams. Mol Syst Biol 4: 211.

15. Shiomi D, Banno S, Homma M, Kawagishi I (2005) Stabilization of polar localization of a chemoreceptor via its covalent modifications and its communication with a different chemoreceptor. J Bacteriol 187: 7647-7654.

16. Sourjik V, Berg HC (2004) Functional interactions between receptors in bacterial chemotaxis. Nature 428: 437-441.

17. Emonet T, Cluzel P (2008) Relationship between cellular response and behavioral variability in bacterial chemotaxis. Proc Natl Acad Sci U S A 105: 3304-3309.

18. Barkai N, Leibler S (1997) Robustness in simple biochemical networks. Nature 387: 913-917.

19. Endres RG, Wingreen NS (2006) Precise adaptation in bacterial chemotaxis through "assistance neighborhoods". Proc Natl Acad Sci U S A 103: 13040-13044.

20. Hansen CH, Endres RG, Wingreen NS (2008) Chemotaxis in *Escherichia coli*: a molecular model for robust precise adaptation. PLoS Comput Biol 4: e1.

21. Vladimirov N, Løvdok L, Lebiedz D, Sourjik V (2008) Dependence of bacterial chemotaxis on gradient shape and adaptation rate. PLoS Comput Biol 4: e1000242.

22. Kalinin YV, Jiang L, Tu Y, Wu M (2009) Logarithmic sensing in *Escherichia coli* bacterial chemotaxis. Biophys J 96: 2439-2448.

23. Li M, Hazelbauer GL (2005) Adaptational assistance in clusters of bacterial chemoreceptors. Mol Microbiol 56: 1617-1626.

24. Berg HC, Purcell EM (1977) Physics of chemoreception. Biophys J 20: 193-219.

25. Bialek W, Setayeshgar S (2005) Physical limits to biochemical signaling. Proc Natl Acad Sci U S A 102: 10040-10045.

26. Bialek W, Setayeshgar S (2008) Cooperativity, sensitivity, and noise in biochemical signaling. Phys Rev Lett 100: 258101.
